# Comparative Analysis of Various Spider Silks in Regard to Nerve Regeneration: Material Properties and Schwann Cell Response

**DOI:** 10.1002/adhm.202302968

**Published:** 2023-12-25

**Authors:** Sarah Stadlmayr, Karolina Peter, Flavia Millesi, Anda Rad, Sonja Wolf, Sascha Mero, Martin Zehl, Axel Mentler, Claudia Gusenbauer, Johannes Konnerth, Hannes C. Schniepp, Helga Lichtenegger, Aida Naghilou, Christine Radtke

**Affiliations:** ^1^ Department of Plastic Reconstructive and Aesthetic Surgery Medical University of Vienna Vienna 1090 Austria; ^2^ Austrian Cluster for Tissue Regeneration Vienna Austria; ^3^ Institute for Physics and Materials Science University of Natural Resources and Life Sciences Vienna 1190 Austria; ^4^ Department of Analytical Chemistry Faculty of Chemistry University of Vienna Vienna 1090 Austria; ^5^ Institute of Soil Research University of Natural Resources and Life Sciences Vienna 1190 Austria; ^6^ Institute of Wood Technology and Renewable Materials University of Natural Resources and Life Sciences Tulln an der Donau 3430 Austria; ^7^ Department of Applied Science William & Mary Williamsburg VA 23185 USA; ^8^ Medical Systems Biophysics and Bioengineering Leiden Academic Centre for Drug Research Leiden University Leiden 2333 The Netherlands

**Keywords:** biomaterials, mechanical properties, migration, morphology, tissue engineering, transcriptomics

## Abstract

Peripheral nerve reconstruction through the employment of nerve guidance conduits with *Trichonephila* dragline silk as a luminal filling has emerged as an outstanding preclinical alternative to avoid nerve autografts. Yet, it remains unknown whether the outcome is similar for silk fibers harvested from other spider species. This study compares the regenerative potential of dragline silk from two orb‐weaving spiders, *Trichonephila inaurata* and *Nuctenea umbratica*, as well as the silk of the jumping spider *Phidippus regius*. Proliferation, migration, and transcriptomic state of Schwann cells seeded on these silks are investigated. In addition, fiber morphology, primary protein structure, and mechanical properties are studied. The results demonstrate that the increased velocity of Schwann cells on *Phidippus regius* fibers can be primarily attributed to the interplay between the silk's primary protein structure and its mechanical properties. Furthermore, the capacity of silk fibers to trigger cells toward a gene expression profile of a myelinating Schwann cell phenotype is shown. The findings for the first time allow an in‐depth comparison of the specific cellular response to various native spider silks and a correlation with the fibers’ material properties. This knowledge is essential to open up possibilities for targeted manufacturing of synthetic nervous tissue replacement.

## Graphical abstract

Nerve guidance conduits prefilled with silk of *Trichonephila* spiders offer a promising alternative to autografts. However, the potential of fibers from other spider species remains unexplored. This study compares regenerative effects of silk from *Trichonephila inaurata*, *Nuctenea umbratica*, and *Phidippus regius*, revealing the influence of silk mechanics on cellular response. This offers novel possibilities for the fabrication of artificial nerve replacements.

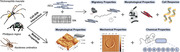

## 1 Introduction

Full functional recovery after peripheral nerve injuries (PNI), evoked by a myriad of causes including traumatic injuries, tumor resection, or iatrogenic damage, is still among the greatest objectives of tissue engineering, nerve reconstructive surgery, and regenerative medicine.^[^ ^1^, ^2^ ^]^ While minor PNIs with mainly intact connective tissue can self‐regenerate via a process orchestrated by pro‐regenerative Schwann cells (SCs), complete nerve transection or nerve defects often result in the loss of sensory, motor, and autonomic function distal to the site of injury, requiring surgical interventions.^[^ ^3^, ^4^, ^5^, ^6^, ^7^ ^]^

After a PNI, autologous nerve grafts, typically harvested from nerves, such as the sural, saphenous, or medial and lateral cutaneous nerve, are considered the current gold standard for treating lost nerve tissue.^[^ ^1^, ^8^, ^9^ ^]^ However, this approach has significant drawbacks, including donor site morbidity, neuroma development, and limited availability of donor tissue.^[^ ^8^ ^]^ Therefore, tissue engineering has focused on developing alternatives to nerve autografts, such as synthetic or biological nerve guidance conduits (NGCs) that can bridge nerve defects while promoting regeneration.^[^ ^10^ ^]^ Although NGCs have shown promising results in repairing noncritical small‐diameter sensory nerves and short‐distance nerve gaps of less than 3 cm, there is still a need for more effective approaches to promote functional restoration for larger‐diameter nerves at longer gaps.^[^ ^8^, ^11^ ^]^ During the initial stages of regeneration within the hollow NGC, the inadequate formation of key extracellular matrix components, such as fibrin, can limit the ingrowth of SCs into the lesion site.^[^ ^12^ ^]^ This can ultimately reduce the essential trophic and topographical guidance structure for regenerating axons, known as bands of Büngner, leading to impaired nerve regeneration through axon dispersion and failed muscle reinnervation.^[^ ^12^, ^13^, ^14^ ^]^ Hence, a longitudinally aligned luminal filler, which protects the NGC from collapsing and functions as a structural guidance scaffold for SCs and axonal sprouts, was used as a strategy to improve the NGC performance and to enhance the microenvironment for regeneration.^[^ ^11^, ^15^ ^]^

One approach to refine the tubular device for nerve regeneration is a prefilling with spider silk fibers, as this biopolymer shows remarkable biocompatibility, toughness, and biodegradability.^[^ ^16^, ^17^ ^]^ A steadily growing number of in vivo and in vitro studies have already demonstrated the outstanding regenerative potential of dragline silk.^[^ ^18^, ^19^, ^20^, ^21^, ^22^, ^23^, ^24^, ^25^, ^26^, ^27^ ^]^ Due to this exceptional success, the field of tissue engineering introduced recombinant proteins that are designed based on native dragline spider silk as a template.^[^ ^28^, ^29^, ^30^ ^]^ However, the majority of the previously deployed silks, whether recombinant or native, resemble dragline silk of the superfamily of orb‐weaving spiders, which represents only 25% of all spider species. Spiders possess diversified and specialized silks with unique physical and biochemical properties suited for the spider's ecological needs.^[^ ^31^ ^]^ Therefore, testing and comparing silks from various species is essential, as specific properties of silks could potentially enhance the regenerative outcome and thus should be considered in the design of advanced templates for recombinant materials. Existing literature has already demonstrated that chemical, morphological, and mechanical properties of biomaterials intricately influence cell behavior, with these factors being interdependent to a certain extent.^[^ ^22^, ^30^, ^32^, ^33^, ^34^, ^35^, ^36^, ^37^ ^]^ Systematically analyzing the isolated effect of individual material parameters, particularly in the case of spider silk, can be challenging. Hence, one strategy for studying the biomaterial's distinct impact on SCs is to compare different spider silks and characterize all potentially relevant properties.

For this reason, in this study, a systematic comparison of the regenerative potential of three spider silks, the dragline silk of two orb‐weaving spider species, *Trichonephila inaurata* and *Nuctenea umbratica*, and the silk of the jumping spider *Phidippus regius*, was conducted. These spiders from different families were specifically chosen to cover a broader range of the silk's properties. For a successful biomedical translation of spider silk fibers, it is fundamental to first analyze and quantify the regenerative effect of silk on cells involved in nerve repair. Therefore detailed in vitro analyses of SCs seeded on spider silk in comparison to SCs cultivated on poly‐*L*‐lysine hydrobromide (PLL)‐laminin coated dishes were performed. Single‐cell tracking via live cell imaging, confocal microscopy of multicolor immunofluorescence‐stained cells, and RNA sequencing were conducted. These experiments pointed out a faster regeneration and by that an enhanced transition of repair SCs toward a myelinating phenotype after 2 weeks of cultivation on silk fibers independent of the spider species. In addition, a significantly higher velocity of SCs on *Phidippus regius* silk was observed, which is a favorable feature for peripheral nerve regeneration. To correlate the detected effects with the characteristics of the silk fibers, we elucidated the fibers’ morphology through atomic force and scanning electron microscopy, their primary protein structure via amino acid analysis and proteomics, and the silks’ mechanical properties by nanoindentation. Our findings suggest that the combination of primary protein structure and mechanical properties could be the drivers behind the fast migration of SCs on silk derived from *Phidippus regius*. This is particularly interesting, as *Phidippus regius* fibers show characteristics of minor ampullate silk, a silk type rarely considered in medical applications. These findings provide valuable insights into the cellular response to various native spider silks and the material properties that drive regeneration, offering the potential for tailored fabrication of nervous tissue replacement in the future.

## 2 Results

### 2.1 Schwann Cells Adhere and Proliferate on All Three Spider Silks

In response to a PNI, SCs undergo a process of dedifferentiation into a specialized repair phenotype. Thereby, SCs execute indispensable steps in the regenerative program and undergo significant changes in their proliferative behavior and morphology to facilitate the restoration of nerve function.^[^ ^38^, ^39^ ^]^ Hence, it is of utmost importance that spider silk as an inner‐lumen NGC filler supports this repair phenotype and allows SC proliferation. Confocal and phase contrast microscopy were used to assess the purity, proliferation, and morphology of SCs.

**Figure 1:**
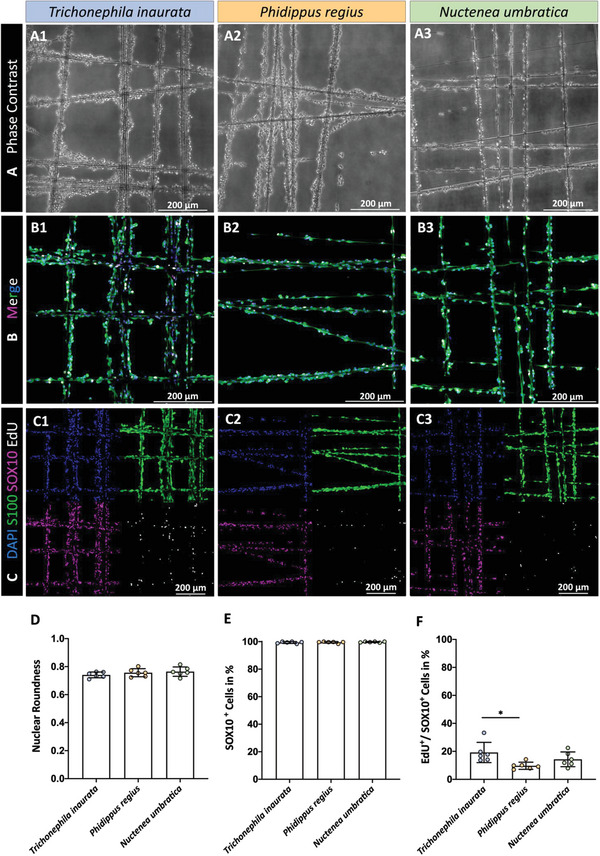
Assessment of the culture purity, cell proliferation, and morphology of rSCs seeded on silk fibers of different species. A) Representative phase contrast micrographs of rSCs after 14 days of cultivation on silk fibers of 1) *Trichonephila inaurata*, 2) *Phidippus regius*, and 3) *Nuctenea umbratica*. B) Merged and C) individual confocal micrographs of rSC cultures on 1) *Trichonephila inaurata*, 2) *Phidippus regius*, and 3) *Nuctenea umbratica* silk fibers stained with DAPI in blue, S100 in green, SOX10 in magenta, and EdU in white. D) Diagram depicting the roundness of rSC nuclei on the silk fibers (mean ± SD, *n* = 6). E) Diagram visualizing the relative amount of SOX10^+^ cells on the silk fibers (mean ± SD, *n* = 6). F) Analysis of proliferating EdU^+^/SOX10^+^ rSCs on the silk fibers (mean ± SD, *n* = 6). *p‐value < 0.05.

For the investigation of cell adhesion and morphology on three spider silk fibers, rat SCs (rSC) were seeded onto the suspended fibers and on a PLL‐laminin coated dish. After incubation for 2 weeks, phase contrast images indicated adherence to all silk fibers (**Figure** **1**A) and to the PLL‐laminin control (Figure S1B, Supporting Information). The cells’ morphology was assessed via the roundness of their DAPI‐stained nuclei and appeared similar without obvious morphological differences on the three fibers (Figure 1B,C,D). Immunofluorescence stainings for the rSC marker SOX10 were performed to assess the SCs’ purity.^[^ ^40^ ^]^ The low number of SOX10^−^/DAPI^+^ cells indicated a rSC culture purity above 99% on different silk fibers (Figure 1B,C,E). The proliferation was quantified via the ratio of EdU^+^/SOX10^+^ rSCs. In general, rSCs were able to proliferate on all silks, whereby a significantly higher rate was observed for the proliferation on fibers of species *Trichonephila inaurata* compared to *Phidippus regius* (Figure 1B,C,F). No significant differences in the proliferation rate were detected between rSCs on silk fibers of *Phidippus regius* and *Nuctenea umbratica*. Overall, all three native spider silk fibers support a proper adhesion and proliferation of rSCs, and do not affect the rSCs’ morphology in different ways. The corresponding data can be found in Table S1 (Supporting Information).

### 2.2 Silk Fibers of *Phidippus regius* Increase the Velocity of Schwann Cells

After a PNI, the distal nerve stump progressively loses its capacity to support the regenerative process due to denervation. Hence, accelerating nerve regeneration is crucial for full functional recovery after PNI.^[^ ^41^, ^42^ ^]^ To that end, efficient migration of SCs toward the injury site, and the accompanied guidance of the regrowing axon, is of critical importance to avoid chronic denervation. Live cell imaging was performed to provide insights into the migratory behavior of SCs on spider silk.

**Figure 2:**
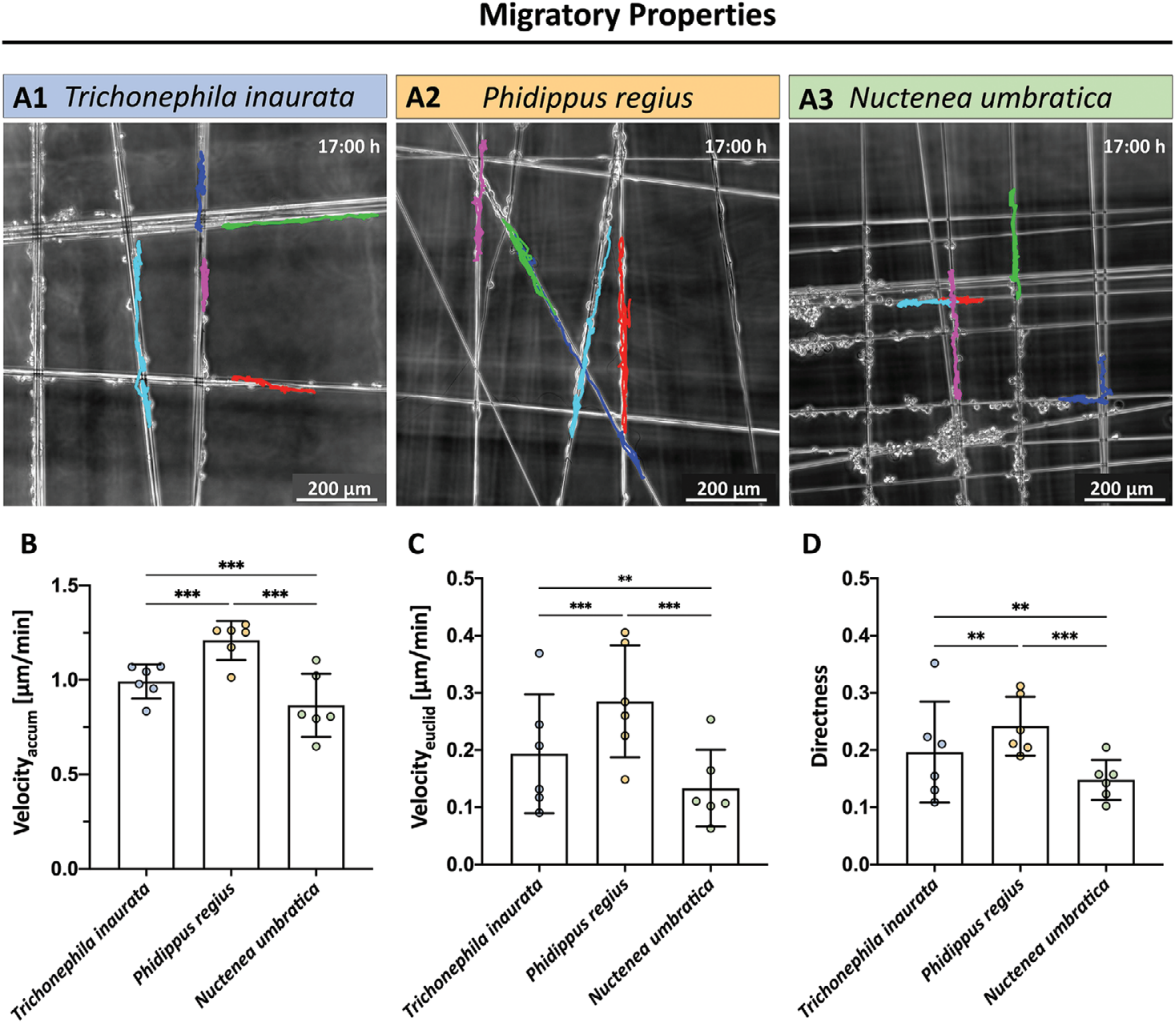
Evaluation of the migratory potential of rSCs on silk fibers of different species. A) Representative micrograph of rSCs after 17 h of live cell imaging on silk fibers of 1) *Trichonephila inaurata*, 2) *Phidippus regius*, and 3) *Nuctenea umbratica*. Each colored line represents an individual rSC's migratory track. B) Quantification of rSCs’ accumulated (total) velocity (Velocity_accum_) in µm min^−1^ on the silk fibers (mean ± SD, *n* = 6). C) Evaluation of rSCs’ Euclidean (effective) velocity (Velocity_euclid_) in µm min^−1^ on the silk fibers (mean ± SD, *n* = 6). D) Diagram depicting the rSCs’ directness on the silk fibers (mean ± SD, *n* = 6). **p‐value < 0.01, ***p‐value < 0.001.

After the initial seeding of rSCs on the three silk fibers, images were obtained every 10 min over a time course of 17 h. **Figure** **2**A depicts phase contrast micrographs of the live cell imaging experiments at the 17 h endpoint. Migratory paths of individual rSCs were tracked throughout the imaging period and were visualized as colored trajectories. While rSCs on silk of *Phidippus regius* showed a significantly higher accumulated (total) and Euclidean (effective) velocity than cells on the other two fibers, cells on fibers of *Nuctenea umbratica* migrated the slowest (Figure 2B,C). All three silk fibers enabled a rSCs migration velocity on the order of 1 mm per 24 h, the average reported axon regeneration rate after a nerve injury.^[^ ^41^ ^]^ Moreover, the directness of the rSCs’ movement on the silks significantly differed, with the highest observed for cells on *Phidippus regius* fibers (Figure 2D). Furthermore, as seen in Figure S1 (Supporting Information), the total velocity and directness of rSCs significantly increased when seeded on silk fibers of *Trichonephila inaurata* and *Phidippus regius* compared to control rSCs cultivated on a PLL‐laminin coated dish. In conclusion, all three silks enable a migration of rSC, however, both the total and effective velocities, as well as the directionality of rSCs on silk fibers of *Phidippus regius* are significantly increased compared to the other conditions. All data are listed in Table S1 (Supporting Information).

### 2.3 All Three Spider Silk Fibers Promote the Transition Toward the Expression Profile of a Myelinating Schwann Cell Phenotype

Upon PNI, SCs dedifferentiate into a proregenerative phenotype thereby executing a repair program that, among others, comprises myelin autophagy, promotes axonal regrowth, and ultimately leads to a remyelination of the regenerating axon.^[^ ^6^, ^7^, ^43^, ^44^, ^45^ ^]^ This axon regeneration followed by the remyelination is of major relevance for a successful recovery. To gain a deeper understanding of the silk‐cell interactions, RNA sequencing was performed after the cultivation of rSCs on spider silk fibers for 14 days to specify the silk‐induced transcriptomic state. This time point was specifically chosen as it represents a later rSC state after the Wallerian degeneration's completion so that the silk's impact on rSCs’ gene expression profile should already be pronounced.^[^ ^46^ ^]^ For comparison, RNA sequencing data of rSCs cultivated on a PLL‐laminin coated dishes was generated after reaching a comparable confluency to cells cultivated on spider silk fibers (Figure S2, Supporting Information).

**Figure 3:**
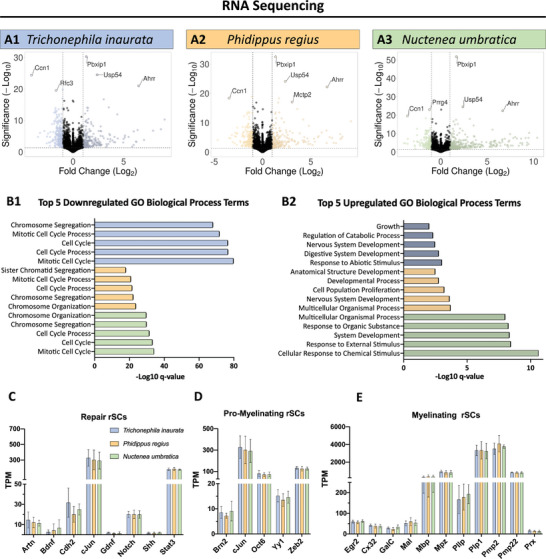
Analysis of the gene expression of rSCs on silk fibers of different species. A) Volcano plots of rSCs after 14 days of cultivation on silk fibers of 1) *Trichonephila inaurata*, 2) *Phidippus regius*, and 3) *Nuctenea umbratica* compared to control rSCs cultivated on a PLL‐laminin coated dish (*n* = 5). B) Enriched biological processes in rSCs cultivated on each silk fiber based on Gene Ontology Biological Process terms for all B1 down‐ and B2 up‐regulated genes of each spider species in contrast to rSCs cultivated on PLL‐laminin. Expression of typical markers for (C) repair rSCs, D) pro‐myelinating rSCs, and E) myelinating rSCs (mean ± SD, *n* = 5).

Volcano plots visualizing the global changes in the transcriptome on each silk fiber are represented in **Figure** **3**A. Upon evaluating the prominent results herein, concordant genes between rSCs cultivated on the three spider silks in comparison to control rSCs were identified. Notably, all these detected genes, namely *Ahrr*, *Usp54*, *Pbxip1*, and *Ccn1*, are known to be associated with the regulation of cell cycle.^[^ ^47^ ^]^ The gene expression analysis disclosed that 485, 454, and 556 genes were differentially expressed in rSCs cultivated on *Trichonephila inaurata*, *Phidippus regius*, and *Nuctenea umbratica*, respectively, compared to the control cells on PLL‐laminin coated dishes. However, the comparison between the individual spider silk fibers revealed no differentially expressed genes. The full list of differentially expressed genes for each condition is available in the Additional File 1. Investigation of the most relevant biological processes influenced by the cultivation of rSCs on silk fibers was conducted with gene ontology (GO) analysis for all upregulated and downregulated genes (Figure 3B, *p*_adj_ < 0.05). The GO terms most strongly associated with upregulated genes among all three silk fibers relate to developmental processes and response to stimuli, while the downregulated processes highlighted a strong reduction in the cell cycle after 14 days in culture. To evaluate the phenotype, and the remyelination capacity of rSCs on silk fibers, transcripts per kilobase million (TPM) values of genes distinct for repair (Figure 3C), pro‐myelinating (Figure 3D), and myelinating rSCs (Figure 3E) were examined. These data revealed that the expression of the genes, which are distinct for the different phenotypes according to literature,^[^ ^7^, ^39^, ^48^, ^49^, ^50^ ^]^ did not vary significantly between rSCs on the three silk fibers. Expression of some myelination‐related genes, such as *Pllp*, and *Plp1*, was increased in rSCs cultured on spider silk fibers in contrast to control cells (Figure S2A, Supporting Information). In addition, when comparing the expression of repair‐ and myelination‐associated genes via the absolute TPM values, the expression of myelination genes of rSCs on silks was at least twice as high as genes typically expressed in repair rSCs (Figure 3C,F). Furthermore, the proliferation of rSCs was slightly reduced after 2 weeks on spider silk relative to the control (Figure S2F, Supporting Information). This finding could be an indication that the rSCs cultivated on spider silk go through an accelerated repair and already adopt a myelinating phenotype after only 2 weeks of cultivation, independent of the spider species.

### 2.4 Silk Fibers Show Comparable Morphology

Due to the evident findings from the RNA‐sequencing results, that the varying rSC velocities are not correlated to transcriptomic changes in the cells, we continued to decipher the reasons behind these detected differences by investigating and comparing the silks’ material properties. Considering the evidence from previous research,^[^ ^32^ ^]^ where it was revealed that the morphology of the substrate has an influence on cells’ velocity, the fibers’ diameter and surface topography were determined with scanning electron, and atomic force microscopy.

**Figure 4:**
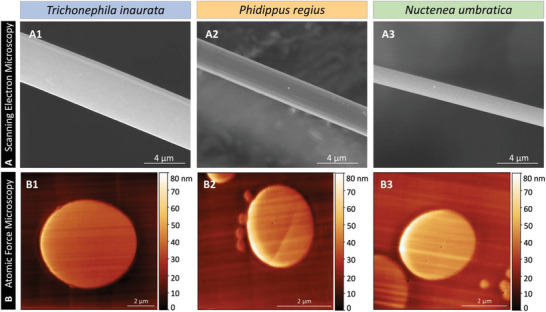
Surface and internal topography of spider silk fibers. A) Scanning electron, and B) atomic force micrographs of the cross‐section silk fibers of 1) *Trichonephila inaurata*, 2) *Phidippus regius*, and 3) *Nuctenea umbratica*.

No visible morphological differences in the silk fibers’ surface were present between the three species based on scanning electron micrographs (**Figure** **4**A). The dragline silk of *Trichonephila inaurata* and *Nuctenea umbratica*, as well as the silk of *Phidippus regius*, appeared smooth and featureless. Evaluation of the scanning electron micrographs exposed differences in the fiber diameter between the species, with the highest average diameter observed for *Trichonephila inaurata* (Figure S3, Supporting Information). Moreover, atomic force microscopy of the fiber's cross‐section displayed no differences between the silks (Figure 4B). Detailed topography images with high magnification captured in the interior of the silk confirmed this observation (Figure S3, Supporting Information). Thus, while all three silks show a similar surface topography and inner morphology, differences in the spider silk diameter can be detected, which, however, do not correlate with the deviating rSC velocity. Detailed data can be found in Table S1 (Supporting Information).

### 2.5 Primary Protein Structure of *Phidippus regius* Fibers Resembles Minor Ampullate Silk

Under in vitro conditions, mimicking the extracellular matrix of the healthy peripheral nerve is usually necessary to ensure appropriate SC attachment, growth, and differentiation. This functionalization of the culture dishes is either done via biopolymers of the peripheral nervous system such as laminin, collagen, or fibronectin, or by short peptide sequences, such as RGD, or IKVAV.^[^ ^30^, ^51^ ^]^ As no preceding modification of the silk surface is required for cell adhesion, we proceeded to conduct a comprehensive analysis of the primary protein structure to elucidate the potential presence of proregenerative motifs within the spidroins. For this we conducted amino acid and proteomic analysis.

The amino acid composition of the dragline silk from *Trichonephila inaurata, Phidippus regius*, and *Nuctenea umbratica* is provided in **Table** **1**. Analysis of the amino acids displayed large variabilities within each spider species, already known from the literature. This is most likely caused by fluctuations in the mixing ratio of the different spidroins in the spinning process.^[^ ^52^ ^]^ In general, the quantitative distribution of the amino acids in the investigated silk fibers was similar, except for threonine, serine, proline, and valine. Furthermore, the quantity of polar, apolar, hydrophobic, and hydrophilic amino acids was almost identical within the three silk fibers. The amino acid content of database‐listed proteins was calculated to verify a proper representation of the amino acid content in our silks compared to the already known spidroins. Whereby, the theoretical and measured amino acid compositions are in good agreement for the three silks (Figure S4A–C, Supporting Information). Moreover, we determined the amino acid composition of *Avicularia avicularia* spider silk as well as the theoretical amino acid composition of silk of the species *Avicularia juruensis*, for the purpose of comparison (Figure S4D, Supporting Information). This comparison is particularly relevant given that the silk of *Avicularia avicularia* was found to be unsuccessful in providing sustained support for SCs over longer time periods as opposed to the three spider silk fibers employed in this study.^[^ ^22^ ^]^ Besides the overall amino acid composition, the proteomic profile of the three spider silk fibers was investigated using liquid chromatography‐mass spectrometry (LC‐MS). Main proteins in the spider silk of *Trichonephila inaurata* (Figure S5, Supporting Information), *Phidippus regius* (Figure S6, Supporting Information), and *Nuctenea umbratica* (Figure S7, Supporting Information) were identified by standard database search and, for highly abundant peptides not assigned in that way, by *de novo* sequencing and homology search. It was confirmed that *Trichonephila inaurata* and *Nuctenea umbratica* possess a spidroin profile already described by Arakawa et al.^[^ ^31^ ^]^ These fibers showed mainly major ampullate spidroins (MaSp). Conversely, the protein profile of *Phidippus regius* revealed a homology or similarity with minor ampullate spidroins (MiSp) (Figure S6, Supporting Information). Based on these examinations it can be deduced that the spider silk fibers harvested from *Phidippus regius* are characterized as MiSp‐like silk, whereby the available sequences of *Phidippus* silk show about 50% overlap with MaSp from the other species (Table S2, Supporting Information).

**Table 1:** Amino acid composition of *Trichonephila inaurata*, *Phidippus regius*, and *Nuctenea umbratica*. Proline values summarize proline and hydroxyproline, as hydroxylation might occur during sample preparation. All values are averages of the individual percentage distribution within one spider species (*n* = 3).

Amino acid analysis			
Amino acid	*Trichonephila inaurata* [wt%]	*Phidippus regius* [wt%]	*Nuctenea umbratica* [wt%]
Asparagine/Aspartic acid	0.85	0.82	0.81
Glutamine/Glutamic acid	6.45	5.15	5.79
Serine	5.60	7.02	5.81
Histidine	0.01	0.02	0.05
Glycine	43.39	43.40	42.55
Threonine	0.46	0.41	1.14
Arginine	0.08	0.04	0.07
Alanine	30.28	28.83	30.14
Tyrosine	5.19	5.60	5.56
Cysteine	2.08	2.18	2.34
Valine	1.31	2.15	1.92
Methionine	0.05	0.01	0.02
Phenylalanine	0.45	0.42	0.45
Isoleucine	0.55	0.68	0.65
Leucine	3.03	2.67	2.38
Lysine	0.03	0.01	0.01
Proline	0.18	0.57	0.31

### 2.6 *Phidippus regius* Silk Exhibits High Hardness and Stiffness

Several studies pointed out the importance of the mechanical properties of biomaterials for numerous cell characteristics, including the guidance of migration, cell adhesion, alignment, proliferation, and phenotype.^[^ ^34^, ^51^ ^]^ Hence, we investigated the silks’ mechanical properties via nanoindentation to elucidate a possible correlation with faster migration on *Phidippus regius*.

**Figure 5:**
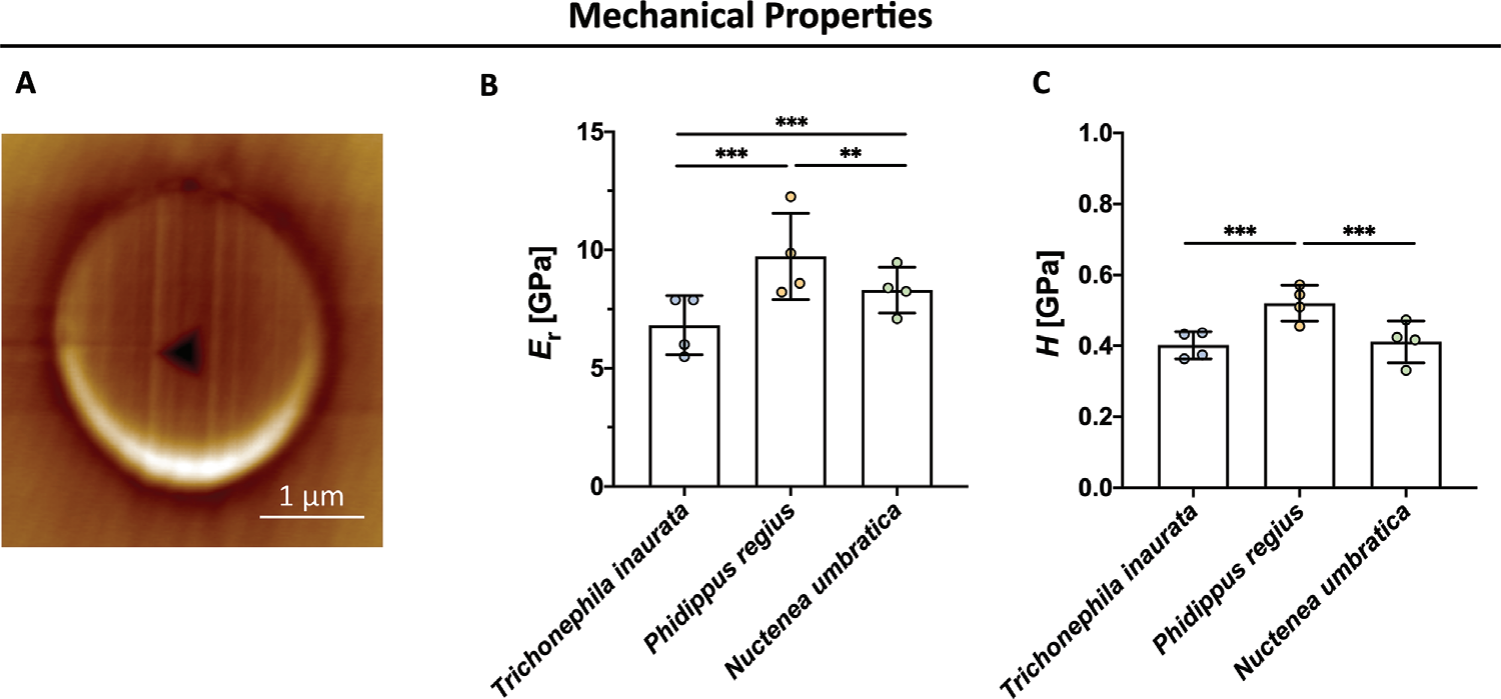
Mechanical properties of spider silk fibers evaluated via nanoindentation. A) Representative SPM micrograph after the indentation of a *Nuctenea umbratica* fiber. B) Analysis of the reduced elastic modulus (*E*_r_) measured in the center of different silk fibers in GPa (mean ± SD, *n* = 4). C) Quantification of the fiber's hardness (H) recorded for the three different species in GPa (mean ± SD, *n* = 4).

To assess the hardness (*H*) and stiffness in a single fiber setup, nanoindentation was performed on the center of cross‐sections of the spider silk fibers after embedding in epoxy resin. A representative scanning probe microscopy (SPM) image after the nanoindentation experiment is shown in **Figure** **5**A. The results of the nanoindentation measurements demonstrated significant differences in the mechanical properties of the fibers derived from three different species. *Phidippus regius* fibers exhibited a significantly higher reduced elastic modulus (*E*_r_) (Figure 5B) and *H* (Figure 5C) in comparison to the silk of the two orb‐weaving spiders. Furthermore, significant differences between the *E*_r_ of *Trichonephila inaurata* and *Nuctenea umbratica* were found, whereas their *H* was nearly identical. Based on the faster migration of rSCs on *Phidippus regius* silk and its significantly higher *E*_r_ and *H*, there could be a relation between the mechanical properties of the fiber and its favorable ability to support higher SC velocities. The data can be found in Table S1 (Supporting Information).

## 3 Discussion

Spider silk has emerged as a highly promising luminal filling material in the field of tissue engineering, particularly for enhancing the microenvironment within NGCs to facilitate peripheral nerve regeneration.^[^ ^19^, ^23^, ^24^, ^26^ ^]^ While previous studies have predominantly focused on investigating the use of dragline silk obtained from the species *Trichonephila* for nervous tissue engineering, less attention has been paid to other spider species’ silk. The search for the ideal NGC‐filling material, regardless of being native or recombinant, is still in progress. Hence, it is essential to expand the repertoire of studied spider silks to elucidate properties of silk crucial for its regenerative potential and to finally pave the way for the successful clinical implementation of silk‐based luminal fillings. This study systematically evaluated the regenerative effect of dragline silk of three spider species and their corresponding morphological, mechanical, and chemical properties. Two orb‐weaving spiders, namely *Trichonephila inaurata* and *Nuctenea umbratica*, and the jumping spider *Phidippus regius* were chosen due to the diverse ecological demands on their silks, which lead to highly specialized silk fibers that cover a range of distinct material properties.

We assessed the success of silks in supporting nerve regeneration through investigation of their effect on SCs in vitro. Following an injury, SCs dedifferentiate into repair cells, characterized by increased proliferation and an elongated phenotype as soon as regeneration tracks are formed.^[^ ^7^ ^]^ All tested spider silks allowed an adhesion and proliferation of SCs, with a morphologically similar SC phenotype. Furthermore, to prevent chronic denervation and enable a sufficient functional recovery, the target structure distal to the injury site needs to be reinnervated within a reasonable time after injury.^[^ ^41^, ^42^ ^]^ SCs were able to use all three fibers as a guidance structure along the silks’ long axis, which opens up the possibility to enhance the regeneration rate not only by navigating SCs but also by directing axonal sprouts of neurons in proximity to the substrate.^[^ ^53^ ^]^ Since SCs on silk fibers of *Phidippus regius* showed a significantly faster and more directed migration, we continued with detailed follow‐up experiments to gain insight into this effect.

As the faster SC migration on silk fibers of *Phidippus regius* might be due to the influence of spider silk on the transcriptomic state of SCs, RNA sequencing was performed after a cultivation of cells on silk fibers for 14 days. Interestingly, silks promoted the expression of genes characteristic of myelinating SCs. Under in vivo conditions, the conversion of repair SCs back to myelinating SCs is observed exclusively following the completion of Wallerian degeneration and subsequent full axonal regeneration.^[^ ^39^ ^]^ This implies that spider silk has the capability to mimic the neuron‐glia interaction thus expediting the nerve's recovery process. Similar findings were reported by previous studies, where it was demonstrated that maturation of SCs is promoted by aligned poly(*ε*‐caprolactone), hyaluronic acid, and poly(lactide‐*co*‐glycolide) electrospun fibers.^[^ ^54^, ^55^, ^56^ ^]^ Although this positive effect was observed, it still cannot be responsible for the measured velocity differences on silks in our study, as no differentially expressed genes were detected between cells incubated on the different silks.

Hence, we continued elucidating the reason for the different velocities by analysis of the silks’ morphology, as the effect of fiber size on migration was formerly shown within certain diameter ranges.^[^ ^32^, ^33^ ^]^ Based on our scanning electron and atomic force micrographs, no differences in the silks’ morphology, surface topography, and internal structure were detected, while variations in the average diameter between fibers of the three species were observed. However, we consider this factor to have only a negligible influence on SC motility, as Wen and Tresco^[^ ^33^ ^]^ have shown a similar cellular migration behavior on filaments in the range of 5–100 µm. Therefore, the smaller fiber diameter variation of 1.6–6.1 µm measured in this project is unlikely to have a notable impact.

Another phenomenon described to influence cells is haptotaxis, whereby directional movement can be affected by surface‐bound cues. In this regard, different signals have been postulated including specific amino acid sequences, such as RGD, and the laminin‐derived motifs IKVAV and YIGSR.^[^ ^30^, ^34^ ^]^ The biofunctionalization with these peptides allows the mimicry of the healthy nerve's extracellular matrix and consequently the enhancement of the microenvironment of SCs in vitro.^[^ ^51^ ^]^ Due to the adherence of SCs on native silk without any preceding coating, we hypothesized that the primary protein structure of our silks could enclose species‐specific haptotactic cues that contribute to the observed migratory effects. Our analysis revealed that the fibers exhibit a striking similarity in their amino acid composition. Furthermore, the measurement of the amino acid content revealed a lower proline content for *Trichonephila inaurata* and *Nuctenea umbratica* compared to the literature,^[^ ^52^, ^57^ ^]^ suggesting that the major portion of the measured silk fibers of both species is rather composed of MaSp1 in comparison to MaSp2. Proteomic analysis of the spider silks revealed a prominent difference between *Phidippus regius* and the two orb‐weavers, with fibers of the jumping spider predominantly aligning with MiSp. It is of note that silk derived from *Phidippus regius* not only shows high homology to MiSp silk but also exhibits a similar to MaSp, highlighting the intriguing and previously described ambiguous differentiation between MaSp and MiSp within jumping spider species.^[^ ^31^ ^]^ Nevertheless, our study provided the first evidence of the outstanding effect of MiSp‐like silk fibers on the migration speed of SCs.

Based on the observation of differences in the spidroin composition of our silks, the mechanical properties of the three fibers were tested, as the primary protein structure has been found to exert a significant influence on mechanical characteristics.^[^ ^58^ ^]^ Repetitive glycine and alanine motifs are associated with the formation of crystalline structures, imparting exceptional strength to the fiber, while a high serine content is correlated with a lower fiber toughness.^[^ ^58^, ^59^, ^60^ ^]^ As the fibers of the spider *Avicularia* were shown to be unsuitable for supporting SCs in vitro due to their low stiffness in the culture medium,^[^ ^22^ ^]^ we conducted a comparison of the amino acid compositions of both successful and unsuccessful spider silks. Our analysis revealed that the three silks examined in this study exhibit a notably higher glycine and a lower content of serine in comparison to the silk of *Avicularia* species. This is a strong indication, that the silk's amino acid composition and its effects on the mechanical properties may play an imperative role in influencing the migration of SCs. Recent studies have already examined the mechanosensitivity of SCs and investigated the effect of the substrate's mechanical characteristics on SC behavior and function. Gu et al. presented that even minor variations in the substrate's mechanical properties can significantly affect the behavior of SCs.^[^ ^35^ ^]^ Yet, the findings remain inconclusive and at times contradictory and no definite correlation between only the mechanical properties and the SCs’ response has been identified so far.^[^ ^35^, ^36^, ^37^, ^61^ ^]^ We demonstrated a significantly higher *E*_r_ and *H* of *Phidippus regius* silk compared to the fibers of the orb‐weaver species. Likewise, we found that *Phidippus regius* silk gave rise to the largest velocities of SCs, which would suggest that better mechanical performance gives rise to higher cell mobility. In comparison, we found smaller differences between the velocities of SCs on fibers of *Trichonephila inaurata* and *Nuctenea umbratica* and the silks’ mechanical properties. However, no consistent trend could be detected between the fibers of these two spider species. Hence, akin to previous research, we could show that mechanical properties alone may not account for the accelerated migration of SCs. Nevertheless, we demonstrated distinct variations between the primary protein structure of *Phidippus regius* and the two orb‐weaving spiders. Considering these facts, we suggest that the enhanced migration velocity of SCs on *Phidippus regius* is influenced by an interplay of the primary protein structure and the mechanical parameters analyzed in our study.

Since our results strongly indicate that the MiSp‐like silk of *Phidippus regius* exhibits inherent advantages for nerve regeneration, this silk type has the potential to become a major focus in tissue engineering. To the best of our knowledge, this marks the first instance of medically oriented research centered around MiSp‐like silks. The finding of accelerated SC migration on MiSp‐like silk, despite subtle differences in its chemical composition and mechanical properties, could carry profound implications of major enhancements in nerve regeneration. Our study demonstrates that the search for the ideal silk template is ongoing, as even minor adjustments in the amino acid sequence and mechanical characteristics of future recombinant silks could enable the reconstruction of significantly longer nerve gaps via fiber‐filled NGCs. Hence, it is crucial to thoroughly explore the potential of novel native fibers to be used as templates for the design of recombinant silk. Consequently, in pursuit of clinical applicability, similar mechanistic studies comparable to the one presented here must be conducted prior to assessing these advanced recombinant silks by in vivo animal experiments.

Although our findings highlight the pivotal role of fiber chemistry and mechanics, it should be noted that alterations in the natural surface coating of silks due to the adsorption of proteins from cell culture media or body fluids, could potentially modify the fiber properties and consequently impact SCs’ behavior. These alterations within the body's biological environment are mainly attributed to the adsorption of host proteins including cytokines, vasoactive agents, and plasma proteases, which in turn are linked to the inherent material properties of silk such as its charge, morphology, and degradation rate.^[^ ^62^, ^63^ ^]^ Hence, it is essential to reevaluate silks, both native and recombinant, subsequent to in vivo experiments to enable more decisive conclusions on the fate of silks’ properties within the body. This is particularly crucial for the emerging coating layer formed on the surface of silk by natural blood proteins and its interactions with surface chemistry of fibers.

## 4 Conclusion

A direct comparison of the morphological, mechanical, and chemical material properties, coupled with comprehensive in vitro experiments conducted in this study highlights the importance of primary protein structure and by that the mechanical properties in native spider silk for its remarkable potential in nerve regeneration. Herein, silk fibers might emulate the characteristics of a bridged axonal lesion, thereby accelerating the regeneration and a redifferentiation of the regenerative into a myelinating SC phenotype after just 2 weeks of cultivation on silk fibers. Furthermore, considering the highly promising outcomes observed for MiSp‐like *Phidippus regius* fibers, it is inevitable for future research in tissue regeneration, particularly in the quest for synthetic nervous tissue replacements, to delve deeper into the effects of minor ampullate silk on cell behavior and function. With regard to clinical translation, our findings introduce new invaluable insights into the options for luminal improvement of NGCs, especially in long‐segment nerve defects.

## 5 Experimental Section

### Harvest of Spider Silk

*Trichonephila inaurata*, *Phidippus regius*, and *Nuctenea umbratica* were kept in individual terraria with optimal conditions for each species. The spiders were fed crickets (*Acheta domesticus*) twice a week and webs were misted with water regularly. Silk was collected by forcibly silking adult female spiders at a speed of 45 cm min^−1^, similar to procedures described previously.^[^ ^25^ ^]^ During the process of harvesting, silk was reeled on a 3D‐printed silk holding frame, fitting into a 4‐well chamber µ‐slide (ibidi), with 15 rotations on each axis. Before seeding cells, sterilization was performed via UV radiation according to Naghilou et al. as this method hardly alters the silk properties.^[^ ^64^ ^]^ Thereby the silk frames were placed at the same distance from the Hg lamp (Osram) and were irradiated for 40 min with a wavelength of 254 nm and a radiated power of 12 W.

For evaluation of material characteristics, silk was harvested with the same procedure and wrapped around a 1 cm^2^ metal frame bent from stainless steel wire (Remanium, Ø 0.70 mm).

### Isolation of Primary Rat Schwann Cells and Cell Culture

Sciatic nerves were harvested from adult male and female Sprague Dawley rats. All animals were sacrificed according to the Austrian Animal Testing Law (TVG 2012, §2, 1.c) and Article 3 of the Directive 2010/63/EU of The European Parliament and of the Council on the Protection of Animals Used for Scientific Purposes.^[^ ^65^ ^]^ The rSCs were isolated and cultured according to well‐established protocols.^[^ ^22^, ^43^ ^]^ Briefly, the pulled nerve fascicles were digested overnight in Minimum Essential Medium ∝ (GIBCO) supplemented with 10% fetal calf serum (LINARIS), 1% penicillin‐streptomycin (GIBCO), 1% sodium pyruvate solution (GIBCO), 2.5% HEPES (Sigma‐Aldrich), 0.125% w/v collagenase type IV (GIBCO), 1.25 U mL^−1^ dispase II (Sigma‐Aldrich), and 3 mm calcium chloride (Merck) at 37 °C and 5% CO_2_. The cell suspension was seeded on culture dishes coated with 0.01% PLL (Sigma‐Aldrich) and 4.8 µg mL^−1^ laminin (Sigma‐Aldrich), whereby rSCs were cultured in Schwann cell expansion medium.^[^ ^21^ ^]^ rSCs were purified by means of a two‐step enrichment protocol to separate them from fibroblasts.^[^ ^66^ ^]^ Cells were passaged upon reaching 80% confluency and were used between passages four to six. After the rSCs reached an adequate purity, they were cryopreserved in Cryo‐SFM medium (PromoCell).

For experiments, cryopreserved rSCs were thawed and cultured as previously described.^[^ ^21^ ^]^ During the seeding process, 1 × 10^5^ cells were placed in a drop of 12.5 µL on the frame with suspended spider silk and kept at 37 °C and 5% CO_2_ for 1 h. Subsequently, the cells were submerged in the culture medium. Furthermore, 1 × 10^4^ cells were seeded as a control on a PLL‐laminin coated 4‐well chamber µ‐slide (ibidi). rSC cultures of six independent donors were used for all in vitro experiments. Phase contrast images were obtained daily with 10×/0.25 or 20×/0.40 objectives of a NIKON Eclipse Ts2R optical microscope.

### Immunofluorescence Staining

Following the cultivation of the rSCs for 14 days, 10 µMm 5‐ethynyl‐2′deoxyuridine (EdU, Invitrogen) was added to the culture for 24 h to assess the culture's proliferation rate. Thereafter rSCs were fixated and stained based on published protocols, whereby the EdU detection was performed using Click‐iT Plus EdU Alexa Fluor 555 imaging kit (Invitrogen) according to the manufacturer's protocol prior to immunofluorescence staining.^[^ ^21^, ^67^ ^]^ The staining panel for the characterization of the rSCs’ purity included the rSC marker S100 and SOX10, and the nuclei stain DAPI. The used primary and secondary antibodies are listed in Table S3 (Supporting Information). For the quantitative analysis of the rSCs’ purity and proliferation, images of the cells were taken with a confocal laser‐scanning microscope (Leica SP8X, or Nikon ECLIPSE T*i*). Hereafter the micrographs were manually assessed, and a minimum of 300 cells positive for DAPI (DAPI^+^) per donor and condition were taken under consideration. Discrimination of rSCs from fibroblasts was based on positive stainings for S100 (S100^+^) and SOX10 (SOX10^+^). To determine the number of proliferating rSCs in culture, EdU^+^/SOX10^+^ rSCs were determined.

Furthermore, for the assessment of the cells’ morphology, the roundness of the nuclei was quantified from DAPI stainings. At least 100 cells of each donor per condition were automatically evaluated using Fiji's Analyze particles macrofunction leading to 600 analyzed cells per condition.

### Live Cell Imaging

Live cell imaging started 3 h after rSC seeding and was performed with an Olympus IX83 microscope using a 10×/0.3 objective. The built‐in stage‐top incubator guaranteed stable conditions at 37 °C and 5% CO_2_ for 17 h of imaging. During this time a phase contrast image was recorded every 10 min using the cellSens software (Version 3.2, Olympus) from multiple preset positions of each condition. The resulting video was manually evaluated using Fiji (Version 2.9.0), and 30 rSCs per donor (*n* = 6) were chosen and tracked via the *Manual Tracking* Fiji plugin achieving 180 tracked cells per condition. Thereby, only cells continuously present in the recording frame throughout the entire 17 h recording were included to calculate the average velocity of rSCs, accounting for potential speed changes over time. For each cell, migratory parameters like directness, as well as effective, and total velocity were determined with the *ibidi Chemotaxis and Migration Tool* Fiji plugin. To correct for movements of the printed frame, the frame was tracked analogous to the cells and afterward subtracted from the measured cells’ migratory values employing a customized Mathematica (Version 12, Wolfram) evaluation algorithm.

### RNA Sequencing and Data Processing

After 14 days of cultivation, rSCs on silk were washed with 1 × PBS, afterward submerged in Monarch DNA/RNA Protection Reagent (New England Biolabs), and stored at −80 °C until RNA isolation. In addition, rSCs seeded on a PLL‐laminin coated 4‐well chamber µ‐slide (ibidi), were treated analogously to rSCs on silk after 4 days in culture when reaching comparable confluency to cells on silk. Five biological replicates per condition were prepared for RNA isolation and subsequent sequencing (*n* = 5). For the isolation, the samples were thawed, and the RNA was extracted using the Monarch Total RNA Miniprep Kit (New England Biolabs) including on‐column DNA digestion with RNase‐free DNase. Sequencing libraries from the total RNA of the samples were prepared using the NEBNext Single Cell/Low Input RNA Library Prep Kit for Illumina (New England Biolabs) according to the manufacturer's instructions. Libraries were quality‐checked on a Bioanalyzer 2100 (Agilent Technologies) using a High Sensitivity DNA Kit for correct insert size and quantified using Qubit dsDNA HS Assay (Invitrogen). Pooled libraries were sequenced on a NextSeq500 instrument (Illumina) in 1 × 75 bp single‐end sequencing mode. Libraries generated an average of 23 million reads per sample.

Reads in FASTQ format were created using the Illumina bcl2fastq command line tool (Version 2.19.1.403, Illumina). FASTQ files were aligned using STAR (Version 2.6.1a) in two‐pass mode^[^ ^68^ ^]^ to Rnor 6.0 reference genome (Version 109.72) and TPM counts were generated by RSEM (Version 1.80).^[^ ^69^ ^]^ Differentially expressed genes were determined using DESeq2 (Version 1.36) package within the R environment.^[^ ^70^, ^71^ ^]^ Visualization of the differences via volcano plots was performed with VolcaNoseR.^[^ ^72^ ^]^ The function of consistently upregulated or downregulated genes was verified via the GeneCards database.^[^ ^47^ ^]^ Enrichment analysis was carried out for genes with a log2FC of at least 1 or maximal −1, and an adjusted *p*‐value of < 0.05 using g:Profiler for Gene‐Ontology Biological Process.^[^ ^73^ ^]^

### Scanning Electron Microscopy

The surface morphology and diameter of silk harvested from *Trichonephila inaurata*, *Phidippus regius*, and *Nuctenea umbratica* were investigated on single silk fibers using a scanning electron microscope with a secondary electron detector (Zeiss Supra 55 VP). A gold layer of 10 nm was coated onto the fibers with a sputter coater equipped with a quartz microbalance for monitoring the layer thickness (Leica SDC050). Micrographs of three randomly chosen positions on each sample were imaged, and the silk diameters were determined using the software SmartTiff (Version 1.0.1.2, Zeiss). Three different spider individuals of each species were evaluated, leading to an average diameter of nine measurements.

### Atomic Force Microscopy

Spider silk fibers of the three species were carefully aligned, clamped in flat embedding molds (Polysciences), and gently submerged in epoxy resin. Subsequently, the epoxy resin was left to dry for 2 days at 60 °C. Epoxy‐embedded silk blocks were freshly cut with an ultramicrotome (Leica RM 22 359) using a histo‐diamond knife (Diatome) for atomic force measurements. Upon slicing the surface, the epoxy blocks were mounted on metallic sample holders using a two‐part epoxy adhesive (UHU). Under ambient conditions, the cut surfaces of the epoxy blocks were studied in ScanAsyst Mode using a Dimension Icon atomic force microscope (Bruker). The surface of the sample was scanned with a ScanAsyst‐Air silicon nitride probe (Bruker) with a nominal spring constant of 0.4 N m^−1^ and a nominal tip radius of 2 nm. Exact spring constants of each atomic force microscopy probe were acquired by the thermal tune method and the deflection sensitivity was calibrated on a sapphire sample. For the resolution of overview scans 1024 × 1024 pixel with a scan rate of 0.3 Hz were chosen, for detailed micrographs with 256 × 256 pixels were recorded. Scans were post‐processed employing the software Gwyddion (Version 2.61, GNU General Public License). Second‐order polynomial functions were applied for background correction and alignment of rows.

### Amino Acid Analysis

An automated online derivatization method for amino acid determination was used for the comparison of various spider silk fibers. Therefore, natural silk samples of three individuals of each spider species (*Trichonephila inaurata*—1 mg, *Phidippus regius*—200 µg, and *Nuctenea umbratica*—200 µg) were hydrolyzed in 1 mL of a 6 Mm hydrochloric acid for 16 h at 115 °C. The automated precolumn derivatization was based on published methods, whereby primary amino acids were derivatized with o‐phthalaldehyde (10 mg mL^−1^), and secondary amino acids were achieved with 9‐fluorenylmethyl chloroformate (2.5 mg mL^−1^) in 0.4 Mm borate buffer.^[^ ^74^, ^75^ ^]^ Amino acid analyses were performed on a 1260 Infinity II LC System (Agilent Technologies) equipped with a binary pump (G1312B), a high‐performance automatic liquid sampler (G1367D), a thermostated column compartment (G1316A), a diode array detector (G4212B), and a fluorescence detector (G1321A). Chromatographic separation was achieved with the ZORBAX Eclipse Plus C18 column (Agilent Technologies, 1.8 µm, 95 Å), and a gradient using eluant (A) water with 10  mMm NaH_2_PO_4_ and 10 mMm Na_2_B_4_O_7_, and eluant (B) Methanol:ACN:milli‐Q water (45:45:10, v:v:v). A flow rate of 0.3 mL min^−1^, a column oven temperature of 40 °C, and an injection volume of 1 µL were applied. Agilent's standard program ChemStation was employed for the identification of amino acids and their analysis. Primary amines were monitored with a diode array detector at a wavelength of 338 nm and a fluorescence detector with an emission of 450 nm (excitation 340 nm), while secondary amino acids were detected at 262 nm and at an emission of 325 nm (excitation 260 nm). The weight percentage distribution of amino acids was calculated out of the average chromatogram peak area.

### Proteomic Analysis

The protein extraction of silk from *Trichonephila inaurata* (200 µg), *Phidippus regius* (40 µg), and *Nuctenea umbratica* (40 µg) was executed with 4 m guanidine thiocyanate (GITC, Carl Roth). After the silk fibers were immersed in GITC, the samples were placed at 95 °C for 2 h. Afterward, the reducing agent 1,4‐dithiothreitol (ThermoFisher) was added to reach a concentration of 10 mm and then the samples were incubated at 90 °C for 1 h. Alkylation was performed by adding 15 mm iodoacetamide and incubating for 1 h at room temperature. The extract was diluted with 5 volumes of ammonium bicarbonate (50 mm, Carl Roth) and was digested overnight at 37 °C with trypsin (ThermoFisher) or chymotrypsin (Promega) at a 60:1 ratio of protein to enzyme. The digestion was stopped via the addition of 1% trifluoroacetic acid (TFA, Carl Roth). Digested peptides were dried, resuspended in 0.1% TFA in water (Sigma‐Aldrich), and purified using the Pierce Peptide Desalting Spin Columns (ThermoFisher) according to the manufacturer's instructions. The protein concentration of purified peptides was determined at 205 and 280 nm. Subsequently, the peptides were dried and stored at −80 °C until analysis.

Dried samples were solubilized in water with 2% acetonitrile (ACN) before LC‐MS analysis. Mass spectra were obtained using a dual‐pressure LTQ Orbitrap Velos (ThermoFisher) mass spectrometer, which was operated in a data‐dependent acquisition mode, equipped with a nanoelectrospray ion source (ThermoFisher) and coupled to a UltiMate 3000 nanoUPLC‐system (ThermoFisher). Each extract (5 µL) was injected onto a 75 µm × 2 cm C18 Acclaim PepMap 100 trap column (ThermoFisher, 3 µm, 100 Å) at a flow rate of 10 µL min^−1^ with 2% ACN, 98% water, 0.05% TFA as loading solvent (10 min total for loading and washing). After loading, the trap column was switched in line with the analytical nanocolumn Acclaim PepMap RSCL (ThermoFisher, 75 µm × 50 cm, C18, 2 µm, 100 Å). A multistep gradient of 2–40% solvent B (80% ACN, 20% water, 0.1% formic acid) was used for the peptide elution, followed by a steeper gradient of 40–80% solvent B within 5 min. After washing the column for 5 additional minutes with 80% solvent B, the gradient was returned to 98% solvent A (2% ACN, 98% water, 0.1% formic acid) to equilibrate the column prior to the next sample injection. Flow rate was held at 300 nL min^−1^ and the temperature at 40 °C. The electrospray voltage was set to 2.1 kV and the ion transfer capillary temperature was 300 °C. For detection, MS scans were acquired in positive ion mode in the range from *m/z* 350–1500 at a resolution of 60 000 (at *m/z* = 400). MS/MS scans were conducted by choosing a top 6 method, selecting precursor ions with a charge state of +2 and higher, surpassing an intensity threshold of 4000. CID fragmentation was performed at 35% normalized collision energy and MS/MS spectra were recorded in the orbitrap at a resolution of 15 000 (at *m/z* = 400). The dynamic exclusion for the selected ions was set to 30 s.

The LC‐MS data were converted with the assistance of MSConvertGUI (Version 3.0, ProteoWizard^[^ ^76^ ^]^). The initial identification of proteins was conducted with MaxQuant (Version 2.2.0, RRID: SCR_01 4485^[^ ^77^ ^]^) and ProteinProspector (Version 6.4.2, RRID: SCR_01 4558). The database search included all spidroins in the NCBI protein database (https://blast.ncbi.nlm.nih.gov/Blast.cgi, on January 11, 2022), proteins from the species *Phidippus regius*, and *Nuctenea umbratica* published in the spider silkome database (on November 28, 2022^[^ ^31^ ^]^), as well as common external contaminants, laboratory proteins, and protein standards provided through the Global Proteome Machine Organization (http://www.thegpm.org/crap/index.html). The precursor mass tolerance was set to 200 ppm with a precursor charge range between 1 and 5 and the fragment mass tolerance to 0.3 Da. Search parameters included trypsin or chymotrypsin as an enzyme, with a maximum of three missed cleavage sites allowed, and a minimum of two peptide identifications per protein, at least one unique. One fixed modification was allowed, carbamidomethylation of cysteine, as well as the variable modifications oxidation of methionine, *N*‐terminal acetylation, and the conversion of *N*‐terminal glutamate to pyroglutamate. The main peaks of the chromatogram, which did not have a peptide match after being searched against the databases, were subsequently (partly) sequenced via a de novo approach. All identified peptides were aligned to their source or homologous proteins via Jalview,^[^ ^78^ ^]^ and the constraint‐based multiple alignment tool.^[^ ^79^ ^]^

### Nanoindentation

Silk fibers of all three species were embedded in epoxy resin analogous to atomic force microscopy samples and sawn with an Accutom‐50 (Struers) to epoxy blocks of 8 mm in length. The epoxy blocks were further processed using an ultramicrotome (Leica RM 22 359) equipped with a histo‐diamond knife (Diatome) to smoothen the surface transverse to the fiber direction. After cutting the surface, the epoxy blocks were fixed to 2 mm thick glass microscopy slides (Logitech) via a two‐part epoxy adhesive (UHU) and mounted on the sample holder of the Hysitron TriboIndenter TI900 (Bruker). Before indentation, a possible penetration of epoxy resin into the embedded silk was excluded utilizing Raman spectroscopy. Using the indenter's SPM imaging mode for precise positioning one indent in the center of the fibers was performed. By means of a Berkovich indenter, the fibers were loaded to a peak load of 100 µN, the latter was held for 20 s before unloading took place at a speed of 33 µN s^−1^. Four different individuals of each species were investigated. Hereby, ten fibers per individual were indented leading to 40 indentations for every spider species. The recorded load‐displacement curves were evaluated with the TriboScan Software using the Oliver–Pharr method,^[^ ^80^, ^81^ ^]^ whereby parameters including the *E*_r_ and *H* were determined. Here, *E* of the spider silk fibers, ≈10 GPa, is about two orders of magnitude lower than *E* of the diamond indenter, ≈1140 GPa. Consequently, the deformation of the indenting tip can be neglected.

### Statistical Analysis

Statistical analysis of data was made with Prism 9 (Version 9.0.2, GraphPad). The normal distribution of the data was assessed by quantile–quantile plots, after that a ROUT outlier test (*Q* = 1) was performed. For the in vitro experiments, experimental conditions were compared independently of donors using a successive two‐way ANOVA approach followed by Tukey's all‐pairs comparisons between group means. A one‐way ANOVA succeeded by Tukey's all‐pairs comparisons was applied for the material characterization experiments. For all experiments, an additional nonparametric Kruskal–Wallis test and Dunn's multiple comparison tests were executed to assess the sensitivity and robustness of the data. If the data were not distributed normally, the statistical analysis after the outliner test was carried out by means of the Kruskal–Wallis test and Dunn's post hoc test. All data are presented as mean ± standard deviation and are listed in Table S1 (Supporting Information).

## Conflict of Interest

The authors declare no conflict of interest.

## Author Contributions

Conceptualization: S.S., H.L., A.N., and C.R. Methodology & Investigation: S.S., K.P., S.W., A.M., M.Z., H.S., and A.N. Material: S.S., F.M., A.R., S.M., G.G., and J.K. Software: S.S., M.Z., and A.N. Visualization: S.S. and M.Z. Formal Analysis: S.S. and A.N. Writing & Draft Preparation: S.S. Writing‐Review & Editing: all authors, Project Administration: S.S. and A.N. Supervision: H.L., A.N., and C.R. Funding Acquisition: H.L., A.N., and C.R.

## Supporting information

Supporting Information

Additional File 1 in addition to the supporting information

## Data Availability

The data that support the findings of this study are available in the supplementary material of this article.

## References

[1] S. Ichihara , Y. Inada , T. Nakamura , Injury 2008, 39, 29. 18804584 10.1016/j.injury.2008.08.029

[2] W. A. Palispis , R. Gupta , Exp. Neurol. 2017, 290, 106. 28111229 10.1016/j.expneurol.2017.01.009

[3] H. J. Seddon , Brain 1943, 66, 237.

[4] J. M. Grasman , D. L. Kaplan , Sci. Rep. 2017, 7, 4092. 28642578 10.1038/s41598-017-04460-8 PMC5481420

[5] S. Meyer Zu Reckendorf , C. Brand , M. T. Pedro , J. Hegler , C. S. Schilling , R. Lerner , L. Bindila , G. Antoniadis , B. Knöll , Nat. Commun. 2020, 11, 2123. 32358558 10.1038/s41467-020-15915-4 PMC7195462

[6] P. J. Arthur‐Farraj , M. Latouche , D. K. Wilton , S. Quintes , E. Chabrol , A. Banerjee , A. Woodhoo , B. Jenkins , M. Rahman , M. Turmaine , G. K. Wicher , R. Mitter , L. Greensmith , A. Behrens , G. Raivich , R. Mirsky , K. R. Jessen , Neuron 2012, 75, 633. 22920255 10.1016/j.neuron.2012.06.021 PMC3657176

[7] K. R. Jessen , R. Mirsky , Front. Cell. Neurosci. 2019, 13, 33. 30804758 10.3389/fncel.2019.00033 PMC6378273

[8] W. Z. Ray , S. E. Mackinnon , Exp. Neurol. 2010, 223, 77. 19348799 10.1016/j.expneurol.2009.03.031 PMC2849924

[9] H. J. Seddon , Ann. R. Coll. Surg. Engl. 1963, 32, 269. 13987582 PMC2311558

[10] G. Hussain , J. Wang , A. Rasul , H. Anwar , M. Qasim , S. Zafar , N. Aziz , A. Razzaq , R. Hussain , J.‐L. G. De Aguilar , T. Sun , Int. J. Biol. Sci. 2020, 16, 116. 31892850 10.7150/ijbs.35653 PMC6930373

[11] R. Gaudin , C. Knipfer , A. Henningsen , R. Smeets , M. Heiland , T. Hadlock , Biomed. Res. Int. 2016, 2016, 3856262. 27556032 10.1155/2016/3856262 PMC4983313

[12] W. Daly , L. Yao , D. Zeugolis , A. Windebank , A. Pandit , J. R. Soc. Interface 2012, 9, 202. 22090283 10.1098/rsif.2011.0438 PMC3243399

[13] D. Ceballos , X. Navarro , N. Dubey , G. Wendelschafer‐Crabb , W. R. Kennedy , R. T. Tranquillo , Exp. Neurol. 1999, 158, 290. 10415137 10.1006/exnr.1999.7111

[14] D. Hoffman‐Kim , J. A. Mitchel , R. V. Bellamkonda , Annu. Rev. Biomed. Eng. 2010, 12, 203. 20438370 10.1146/annurev-bioeng-070909-105351 PMC3016849

[15] C. R. Carvalho , J. M. Oliveira , R. L. Reis , Front. Bioeng. Biotechnol. 2019, 7, 337. 31824934 10.3389/fbioe.2019.00337 PMC6882937

[16] F. Schäfer‐Nolte , K. Hennecke , K. Reimers , R. Schnabel , C. Allmeling , P. M. Vogt , J. W. Kuhbier , U. Mirastschijski , Ann. Surg. 2014, 259, 781. 23873006 10.1097/SLA.0b013e3182917677

[17] F. Bergmann , S. Stadlmayr , F. Millesi , M. Zeitlinger , A. Naghilou , C. Radtke , Biomater. Adv. 2022, 140, 213089. 36037764 10.1016/j.bioadv.2022.213089

[18] C. Allmeling , A. Jokuszies , K. Reimers , S. Kall , P. M. Vogt , J. Cell. Mol. Med. 2006, 10, 770. 16989736 10.1111/j.1582-4934.2006.tb00436.x PMC3933158

[19] C. Allmeling , A. Jokuszies , K. Reimers , S. Kall , C. Y. Choi , G. Brandes , C. Kasper , T. Scheper , M. Guggenheim , P. M. Vogt , Cell. Prolif. 2008, 41, 408. 18384388 10.1111/j.1365-2184.2008.00534.x PMC6496660

[20] F. Roloff , S. Strauß , P. M. Vogt , G. Bicker , C. Radtke , Biomed. Res. Int. 2014, 2014, 906819. 24949480 10.1155/2014/906819 PMC4052499

[21] F. Millesi , T. Weiss , A. Mann , M. Haertinger , L. Semmler , P. Supper , D. Pils , A. Naghilou , C. Radtke , FASEB J. 2021, 35, e21196. 33210360 10.1096/fj.202001447R PMC7894153

[22] A. Naghilou , L. Pöttschacher , F. Millesi , A. Mann , P. Supper , L. Semmler , T. Weiss , E. H. G. Backus , C. Radtke , Mater. Sci. Eng. C 2020, 116, 111219. 10.1016/j.msec.2020.111219 32806225

[23] T. Kornfeld , J. Nessler , C. Helmer , R. Hannemann , K. H. Waldmann , C. T. Peck , P. Hoffmann , G. Brandes , P. M. Vogt , C. Radtke , Biomaterials 2021, 271, 120692. 33607544 10.1016/j.biomaterials.2021.120692

[24] C. Radtke , C. Allmeling , K.‐H. Waldmann , K. Reimers , K. Thies , H. C. Schenk , A. Hillmer , M. Guggenheim , G. Brandes , P. M. Vogt , PLoS One 2011, 6, e16990. 21364921 10.1371/journal.pone.0016990 PMC3045382

[25] J. W. Kuhbier , C. Allmeling , K. Reimers , A. Hillmer , C. Kasper , B. Menger , G. Brandes , M. Guggenheim , P. M. Vogt , PLoS One 2010, 5, e12032. 20711495 10.1371/journal.pone.0012032 PMC2918503

[26] L. Semmler , A. Naghilou , F. Millesi , S. Wolf , A. Mann , S. Stadlmayr , S. Mero , L. Ploszczanski , L. Greutter , A. Woehrer , E. Placheta‐Györi , F. Vollrath , T. Weiss , C. Radtke , Adv. Healthcare Mater. 2023, 12, 2203237. 10.1002/adhm.202203237 PMC11468823 36683305

[27] A. Magaz , A. Faroni , J. E. Gough , A. J. Reid , X. Li , J. J. Blaker , Adv. Healthcare Mater. 2018, 7, e1800308. 10.1002/adhm.201800308 30260575

[28] T. B. Aigner , E. Desimone , T. Scheibel , Adv. Mater. 2018, 30, 1704636. 10.1002/adma.201704636 29436028

[29] M. Ramezaniaghdam , N. D. Nahdi , R. Reski , Front. Bioeng. Biotechnol. 2022, 10, 835637. 35350182 10.3389/fbioe.2022.835637 PMC8957953

[30] V. T. Trossmann , T. Scheibel , Adv. Healthcare Mater. 2023, 12, 2202660. 10.1002/adhm.202202660 PMC11468868 36565209

[31] K. Arakawa , N. Kono , A. D. Malay , A. Tateishi , N. Ifuku , H. Masunaga , R. Sato , K. Tsuchiya , R. Ohtoshi , D. Pedrazzoli , A. Shinohara , Y. Ito , H. Nakamura , A. Tanikawa , Y. Suzuki , T. Ichikawa , S. Fujita , M. Fujiwara , M. Tomita , S. J. Blamires , J.‐A. Chuah , H. Craig , C. P. Foong , G. Greco , J. Guan , C. Holland , D. L. Kaplan , K. Sudesh , B. B. Mandal , Y. Norma‐Rashid , et al., Sci. Adv. 2022, 8, eabo6043. 36223455 10.1126/sciadv.abo6043 PMC9555773

[32] S. Gnavi , B. E. Fornasari , C. Tonda‐Turo , G. Ciardelli , M. Zanetti , S. Geuna , I. Perroteau , Mater. Sci. Eng., C 2015, 48, 620. 10.1016/j.msec.2014.12.055 25579965

[33] X. Wen , P. A. Tresco , J. Biomed. Mater. Res., Part A 2006, 76, 626. 10.1002/jbm.a.30520 16287096

[34] M. R. Wrobel , H. G. Sundararaghavan , Tissue Eng., Part B 2014, 20, 93. 10.1089/ten.TEB.2013.0233 23815309

[35] Y. Gu , Y. Ji , Y. Zhao , Y. Liu , F. Ding , X. Gu , Y. Yang , Biomaterials 2012, 33, 6672. 22738780 10.1016/j.biomaterials.2012.06.006

[36] G. Rosso , I. Liashkovich , P. Young , D. Röhr , V. Shahin , Nanomed. Nanotechnol. Biol. Med. 2017, 13, 493. 10.1016/j.nano.2016.06.011 27389149

[37] E. B. Evans , S. W. Brady , A. Tripathi , D. Hoffman‐Kim , Biomater. Res. 2018, 22, 14. 29780613 10.1186/s40824-018-0124-z PMC5948700

[38] K. R. Jessen , R. Mirsky , J. Physiol. 2016, 594, 3521. 26864683 10.1113/JP270874 PMC4929314

[39] A. Balakrishnan , L. Belfiore , T.‐H. Chu , T. Fleming , R. Midha , J. Biernaskie , C. Schuurmans , Front. Mol. Neurosci. 2021, 13, 608442. 33568974 10.3389/fnmol.2020.608442 PMC7868393

[40] M. Haertinger , T. Weiss , A. Mann , A. Tabi , V. Brandel , C. Radtke , Cells 2020, 9, 163. 31936601 10.3390/cells9010163 PMC7016740

[41] W. Sulaiman , T. Gordon , Ochsner. J. 2013, 13, 100. 23531634 PMC3603172

[42] M. Golshadi , E. F. Claffey , J. K. Grenier , A. Miller , M. Willand , M. G. Edwards , T. P. Moore , M. Sledziona , T. Gordon , G. H. Borschel , J. Cheetham , NPJ Regen. Med. 2023, 8, 12. 36849720 10.1038/s41536-023-00285-4 PMC9970988

[43] T. Weiss , S. Taschner‐Mandl , A. Bileck , A. Slany , F. Kromp , F. Rifatbegovic , C. Frech , R. Windhager , H. Kitzinger , C.‐H. Tzou , P. F. Ambros , C. Gerner , I. M. Ambros , Glia 2016, 64, 2133. 27545331 10.1002/glia.23045

[44] J. A. Gomez‐Sanchez , L. Carty , M. Iruarrizaga‐Lejarreta , M. Palomo‐Irigoyen , M. Varela‐Rey , M. Griffith , J. Hantke , N. Macias‐Camara , M. Azkargorta , I. Aurrekoetxea , V. G. De Juan , H. B. J. Jefferies , P. Aspichueta , F. Elortza , A. M. Aransay , M. L. Martínez‐Chantar , F. Baas , J. M. Mato , R. Mirsky , A. Woodhoo , K. R. Jessen , J. Cell. Biol. 2015, 210, 153. 26150392 10.1083/jcb.201503019 PMC4494002

[45] S. Kim , J. C. Maynard , A. Strickland , A. L. Burlingame , J. Milbrandt , Proc. Natl. Acad. Sci. USA 2018, 115, 8019. 30012597 10.1073/pnas.1805538115 PMC6077742

[46] S. Rotshenker , J. Neuroinflammation 2011, 8, 109. 21878125 10.1186/1742-2094-8-109 PMC3179447

[47] G. Stelzer , N. Rosen , I. Plaschkes , S. Zimmerman , M. Twik , S. Fishilevich , T. I. Stein , R. Nudel , I. Lieder , Y. Mazor , S. Kaplan , D. Dahary , D. Warshawsky , Y. Guan‐Golan , A. Kohn , N. Rappaport , M. Safran , D. Lancet , Curr. Protoc. Bioinform. 2016, 54, 1301. 10.1002/cpbi.5 27322403

[48] M. E. Kastriti , I. Adameyko , Curr. Opin. Neurobiol. 2017, 47, 196. 29161639 10.1016/j.conb.2017.11.004

[49] R. Mirsky , A. Woodhoo , D. B. Parkinson , P. Arthur‐Farraj , A. Bhaskaran , K. R. Jessen , J. Peripher. Nerv. Syst. 2008, 13, 122. 18601657 10.1111/j.1529-8027.2008.00168.x

[50] K. R. Jessen , R. Mirsky , Glia 2008, 56, 1552. 18803323 10.1002/glia.20761

[51] L. Lotfi , M. Khakbiz , M. Moosazadeh Moghaddam , S. Bonakdar , J. Biomed. Mater. Res., Part A 2019, 107, 2425. 10.1002/jbm.a.36749 31254439

[52] M. S. Creager , J. E. Jenkins , L. A. Thagard‐Yeaman , A. E. Brooks , J. A. Jones , R. V. Lewis , G. P. Holland , J. L. Yarger , Biomacromolecules 2010, 11, 2039. 20593757 10.1021/bm100399x PMC2922512

[53] C. Miller , S. Jeftinija , S. Mallapragada , Tissue Eng. 2001, 7, 705. 11749728 10.1089/107632701753337663

[54] S. Y. Chew , R. Mi , A. Hoke , K. W. Leong , Biomaterials 2008, 29, 653. 17983651 10.1016/j.biomaterials.2007.10.025 PMC2713097

[55] J. Radhakrishnan , A. A. Kuppuswamy , S. Sethuraman , A. Subramanian , J. Biomed. Nanotechnol. 2015, 11, 512. 26307833 10.1166/jbn.2015.1921

[56] M. R. Wrobel , H. G. Sundararaghavan , Neuroscience 2018, 376, 172. 29462706 10.1016/j.neuroscience.2018.02.015

[57] T. A. Blackledge , M. Kuntner , M. Marhabaie , T. C. Leeper , I. Agnarsson , Sci. Rep. 2012, 2, 833. 23150784 10.1038/srep00833 PMC3495280

[58] H. C. Craig , D. Piorkowski , S. Nakagawa , M. M. Kasumovic , S. J. Blamires , J. R. Soc. Interface 2020, 17, 20200471. 32993436 10.1098/rsif.2020.0471 PMC7536055

[59] O. Tokareva , M. Jacobsen , M. Buehler , J. Wong , D. L. Kaplan , Acta Biomater. 2014, 10, 1612. 23962644 10.1016/j.actbio.2013.08.020 PMC3926901

[60] A. Sponner , W. Vater , S. Monajembashi , E. Unger , F. Grosse , K. Weisshart , PLoS One 2007, 2, e998. 17912375 10.1371/journal.pone.0000998 PMC1994588

[61] Z. Xu , J. A. Orkwis , B. M. Devine , G. M. Harris , J. Tissue Eng. Regener. Med. 2020, 14, 229. 10.1002/term.2987 31702874

[62] M. Widhe , J. Johansson , M. Hedhammar , A. Rising , Biopolymers 2012, 97, 468. 21898363 10.1002/bip.21715

[63] V. T. Trossmann , S. Lentz , T. Scheibel , J. Funct. Biomater. 2023, 14, 434. 37623678 10.3390/jfb14080434 PMC10455157

[64] A. Naghilou , K. Peter , F. Millesi , S. Stadlmayr , S. Wolf , A. Rad , L. Semmler , P. Supper , L. Ploszczanski , J. Liu , M. Burghammer , C. Riekel , A. Bismarck , E. H. G. Backus , H. Lichtenegger , C. Radtke , Int. J. Biol. Macromol. 2023, 244, 125398. 37330085 10.1016/j.ijbiomac.2023.125398

[65] Official Journal of the European Union , European Parliament CotEU. Directive 2010/63/EU of the European Parliament and of the Council of 22 September 2010 on the Protection of Animals Used for Scientific Purposes (Text with EEA Relevance) , 2010.

[66] T. Weiss , S. Taschner‐Mandl , P. F. Ambros , I. M. Ambros , in Schwann Cells. Methods in Molecular Biology, Vol. 1739 (Eds: P. Monje , H. Kim ), Humana Press, New York 2018, pp. 67. 10.1007/978-1-4939-7649-2_5 29546701

[67] F. Millesi , S. Mero , L. Semmler , A. Rad , S. Stadlmayr , A. Borger , P. Supper , M. Haertinger , L. Ploszczanski , U. Windberger , T. Weiss , A. Naghilou , C. Radtke , ACS Appl. Mater. Interfaces 2023, 15, 12678. 36876876 10.1021/acsami.2c20040 PMC10020957

[68] A. Dobin , C. A. Davis , F. Schlesinger , J. Drenkow , C. Zaleski , S. Jha , P. Batut , M. Chaisson , T. R. Gingeras , Bioinformatics 2013, 29, 15. 23104886 10.1093/bioinformatics/bts635 PMC3530905

[69] B. Li , C. N. Dewey , BMC Bioinformatics 2011, 12, 323. 21816040 10.1186/1471-2105-12-323 PMC3163565

[70] M. I. Love , W. Huber , S. Anders , Genome Biol. 2014, 15, 550. 25516281 10.1186/s13059-014-0550-8 PMC4302049

[71] A. Zhu , J. G. Ibrahim , M. I. Love , Bioinformatics 2019, 35, 2084. 30395178 10.1093/bioinformatics/bty895 PMC6581436

[72] J. Goedhart , M. S. Luijsterburg , Sci. Rep. 2020, 10, 20560. 33239692 10.1038/s41598-020-76603-3 PMC7689420

[73] U. Raudvere , L. Kolberg , I. Kuzmin , T. Arak , P. Adler , H. Peterson , J. Vilo , Nucl. Acids Res. 2019, 47, W191. 31066453 10.1093/nar/gkz369 PMC6602461

[74] J. W. Henderson , R. D. Ricker , B. A. Bidlingmeyer , C. Woodward , Agilent Technologies, Application Note, Publication No: 5980–1193, 2000.

[75] J. W. Henderson , A. Brooks , 2010, https://www.agilent.com/Library/applications/5990‐4547EN.pdf.

[76] M. C. Chambers , B. Maclean , R. Burke , D. Amodei , D. L. Ruderman , S. Neumann , L. Gatto , B. Fischer , B. Pratt , J. Egertson , K. Hoff , D. Kessner , N. Tasman , N. Shulman , B. Frewen , T. A. Baker , M.‐Y. Brusniak , C. Paulse , D. Creasy , L. Flashner , K. Kani , C. Moulding , S. L. Seymour , L. M. Nuwaysir , B. Lefebvre , F. Kuhlmann , J. Roark , P. Rainer , S. Detlev , T. Hemenway , et al., Nat. Biotechnol. 2012, 30, 918. 23051804 10.1038/nbt.2377 PMC3471674

[77] J. Cox , M. Mann , Nat. Biotechnol. 2008, 26, 1367. 19029910 10.1038/nbt.1511

[78] A. M. Waterhouse , J. B. Procter , D. M. A. Martin , M. Clamp , G. J. Barton , Bioinformatics 2009, 25, 1189. 19151095 10.1093/bioinformatics/btp033 PMC2672624

[79] J. S. Papadopoulos , R. Agarwala , Bioinformatics 2007, 23, 1073. 17332019 10.1093/bioinformatics/btm076

[80] W. C. Oliver , G. M. Pharr , J. Mater. Res. 1992, 7, 1564.

[81] W. Gindl , J. Konnerth , T. Schöberl , Cellulose 2006, 13, 1.

