# Supplementary material for: Comparative Analysis of Various Spider Silks in Regard to Nerve Regeneration: Material Properties and Schwann Cell Response
Source: Adv Healthc Mater. 2023 Dec 25;13(8):2302968. doi: 10.1002/adhm.202302968 (PMC11468126; doi:10.1002/adhm.202302968)
Supplement: Supplementary file 1 — Supporting Information [file ADHM-13-2302968-s001.pdf]

ADVANCED
HEALTHCARE
MATERIALS

Supporting Information

for *Adv. Healthcare Mater.*, DOI 10.1002/adhm.202302968

Comparative Analysis of Various Spider Silks in Regard to Nerve Regeneration: Material Properties and Schwann Cell Response

Sarah Stadlmayr, Karolina Peter, Flavia Millesi, Anda Rad, Sonja Wolf, Sascha Mero, Martin Zehl, Axel Mentler, Claudia Gusenbauer, Johannes Konnerth, Hannes C. Schniepp, Helga Lichtenegger, Aida Naghilou and Christine Radtke*

Supporting Information for

Comparative Analysis of Various Spider Silks in Regard to Nerve Regeneration: Material Properties and Schwann Cell Response

Sarah Stadlmayr[1,2], Karolina Peter[3], Flavia Millesi[1,2], Anda Rad[1], Sonja Wolf[1], Sascha Mero[1], Martin Zehl[4], Axel Mentler[5], Claudia Gusenbauer[6], Johannes Konnerth[6], Hannes C. Schniepp[7], Helga Lichtenegger[3], Aida Naghilou[1,2,8,*], Christine Radtke[1,2]

[1] Department of Plastic, Reconstructive and Aesthetic Surgery, Medical University of Vienna, Vienna, Austria

[2] Austrian Cluster for Tissue Regeneration, Vienna, Austria

[3] Institute for Physics and Materials Science, University of Natural Resources and Life Sciences, Vienna, Austria

[4] Department of Analytical Chemistry, Faculty of Chemistry, University of Vienna, Austria

[5] Institute of Soil Research, University of Natural Resources and Life Sciences, Vienna, Austria

[6] Institute of Wood Technology and Renewable Materials, University of Natural Resources and Life Sciences, Vienna, Austria

[7] Department of Applied Science, William & Mary, Virginia, United States

[8] Medical Systems Biophysics and Bioengineering, Leiden Academic Centre for Drug Research, Leiden University, Leiden, The Netherlands

* corresponding author: aida.naghilou@meduniwien.ac.at

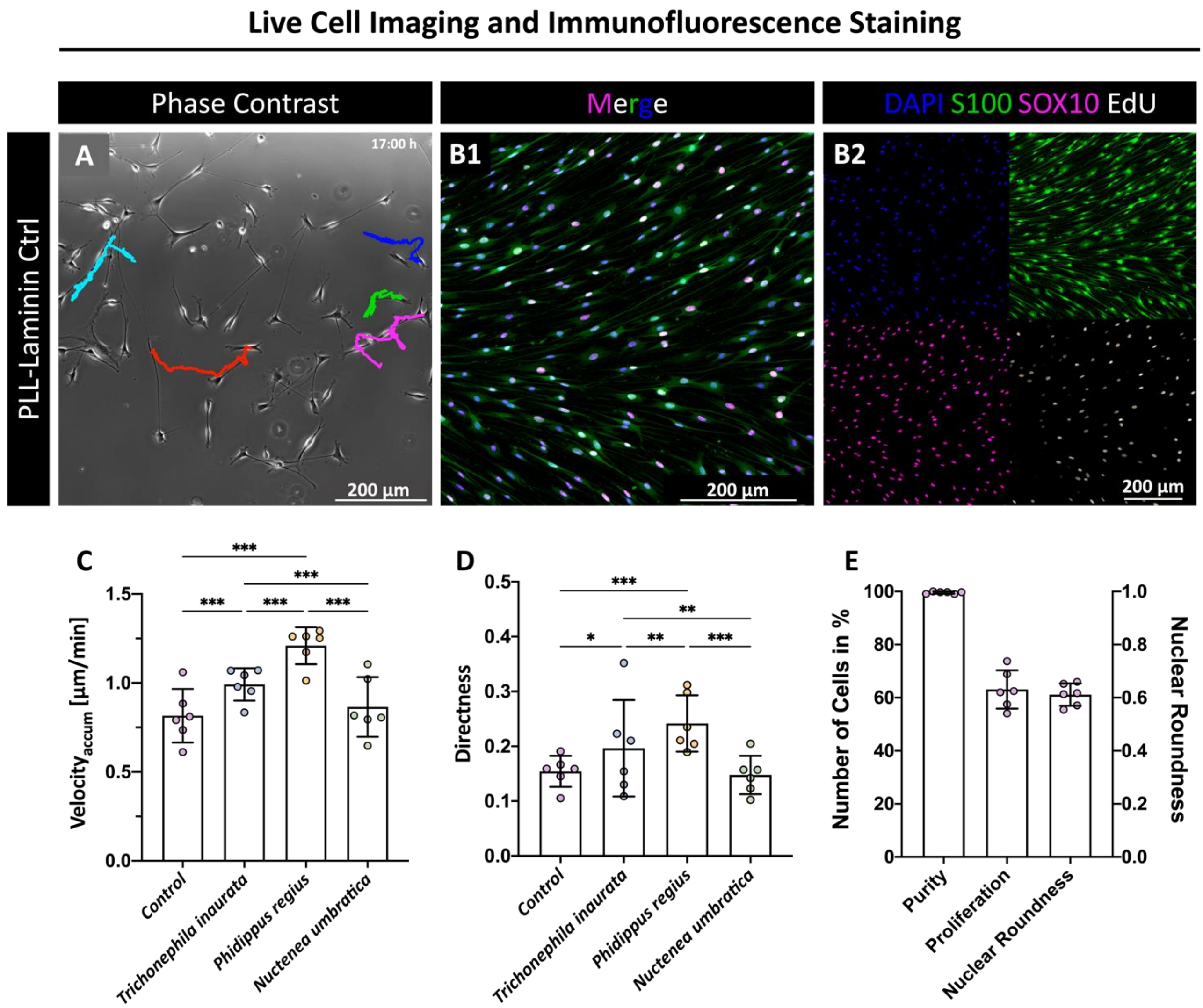

Figure S1: **Assessment of the migratory potential, purity, proliferation, and morphology of rSCs seeded on a PLL-laminin coated dish (Ctrl) in comparison to the three spider silk fibers**. **A** Representative phase contrast micrograph of rSCs after 17 hours of live cell imaging on the control. Each colored line represents an individual rSC's migratory track. **B1** Merged and **B2** individual confocal micrographs of rSC cultures stained with DAPI in blue, for S100 in green, SOX10 in magenta, and EdU in white. **C** Quantification of rSCs' accumulated (total) velocity in µm/min and **D** directness of rSCs (mean ± SD, *n*=6) seeded on four different conditions (Ctrl, *Trichonephila inaurata, Phidippus regius*, and *Nuctenea umbratica*). **E** Diagram visualizing SOX10$^+$ rSCs, EdU$^+$/SOX10$^+$ cells, and the nuclear roundness of rSCs on the control (mean ± SD, *n*=6). * *p*-value < 0.05, ** *p*-value < 0.01, *** *p*-value < 0.001.

RNA Sequencing

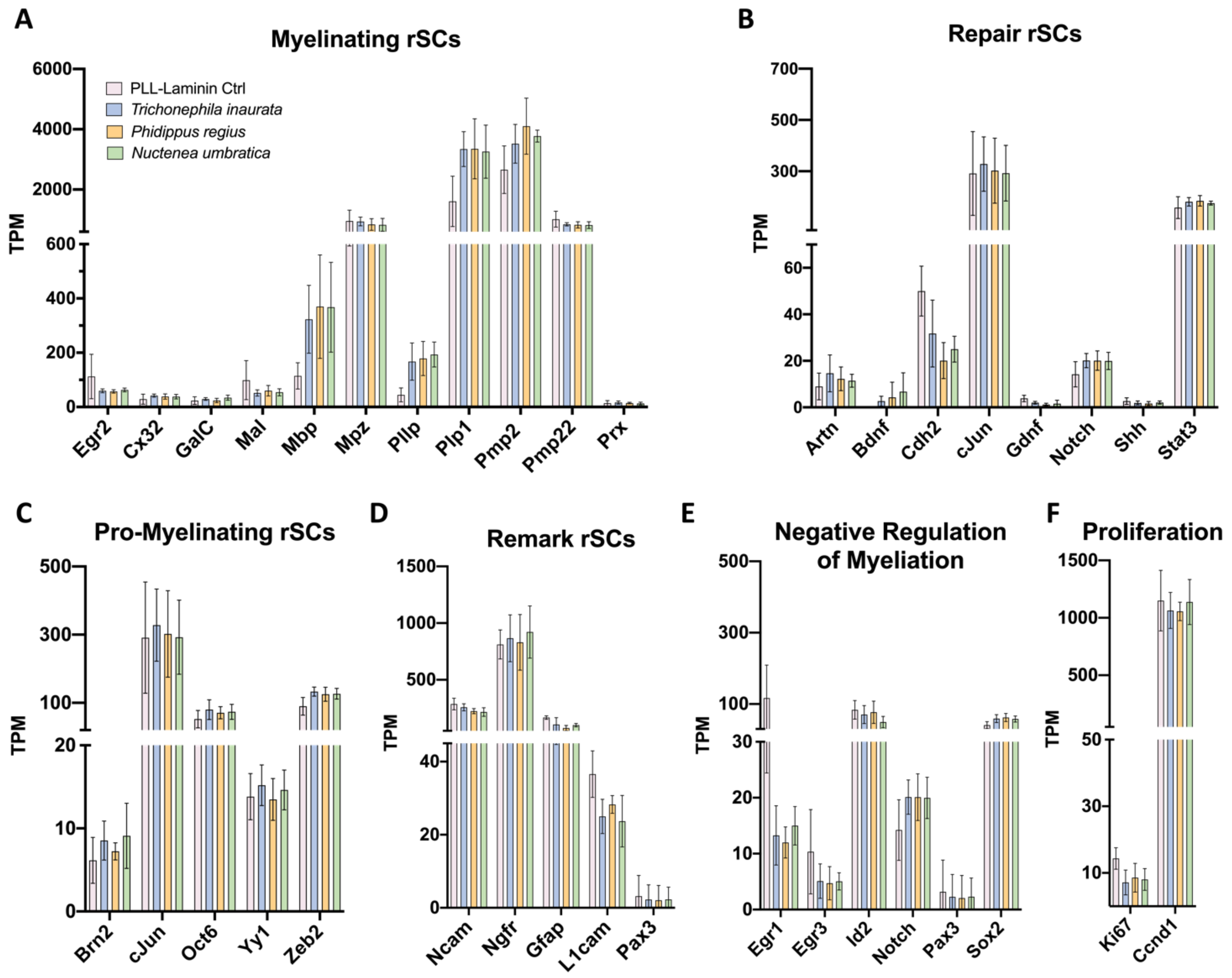

Figure S2: Analysis of the gene expression of rSCs on a PLL-laminin control (Ctrl) and spider silk fibers of different species. Expression of typical markers for A myelinating rSCs, B repair rSCs, C pro-myelinating rSCs, D Remark rSCs, E negative regulators of myelination, and F proliferation (mean ± SD, *n*=5).

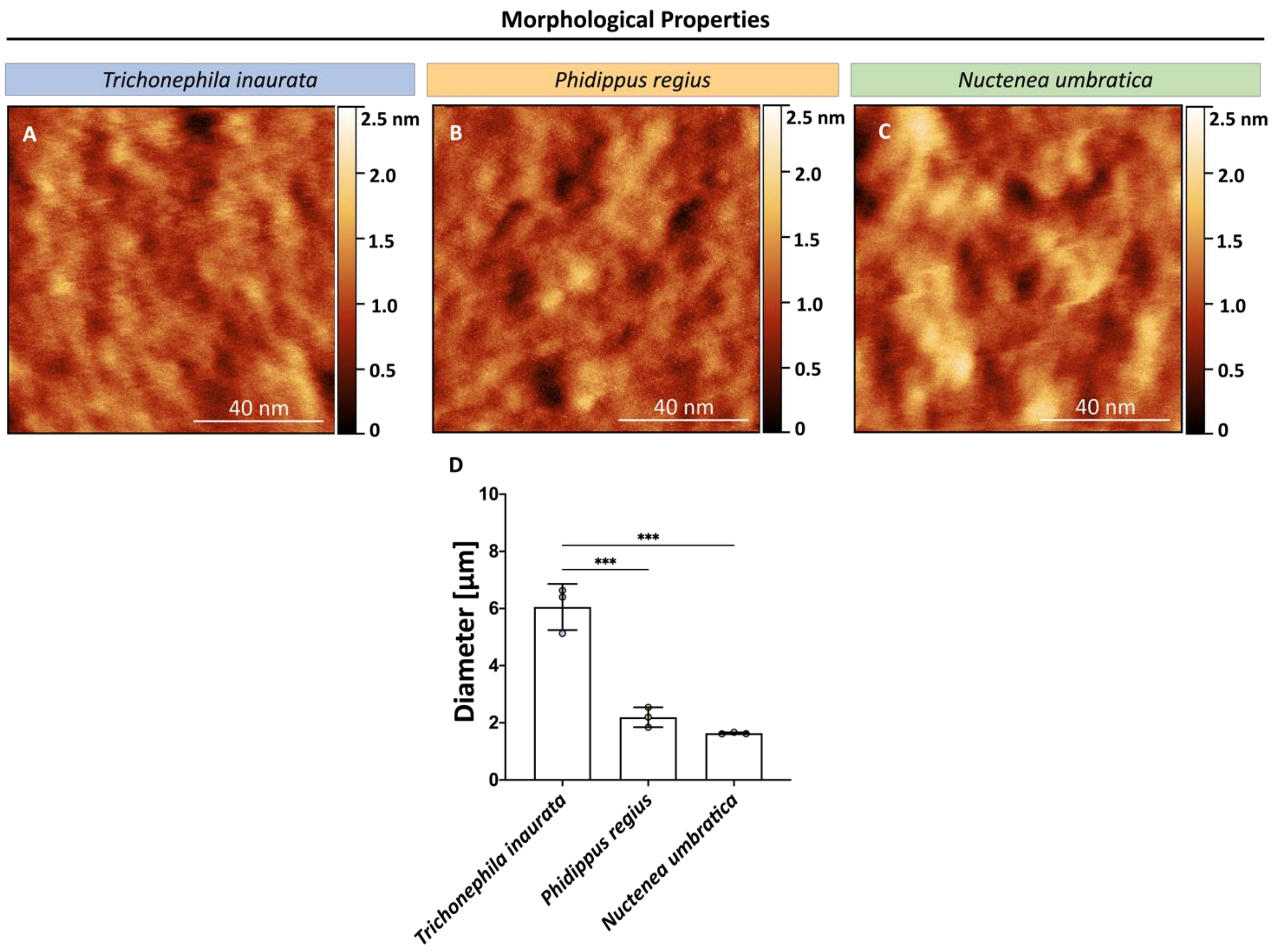

Figure S3: **Detailed atomic force micrographs and average diameter of three spider silk fibers**. The internal topography of **A** *Trichonephila inaurata*, **B** *Phidippus regius*, and **C** *Nuctenea umbratica* was imaged in the center of the silk fibers. **D** Quantification of the spider silk diameter determined via scanning electron images (mean ± SD, n=3). *** p-value < 0.001.

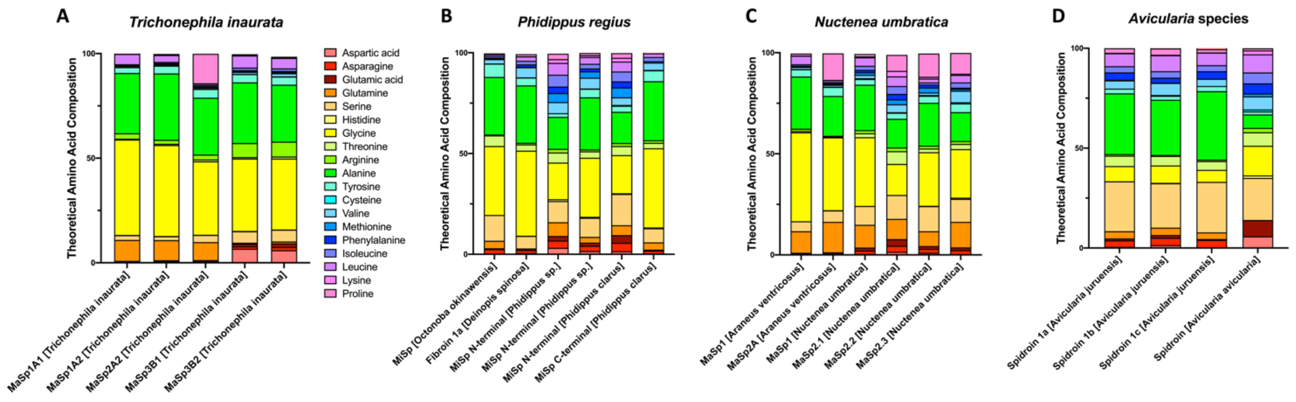

Figure S4: **Theoretical amino acid composition calculated out of database proteins found in *Trichonephila inaurata*, *Phidippus regius*, *Nuctenea umbratica,* and *Avicularia* species silk samples.** The amino acid percentage distribution for proteins listed in the NCBI or silkome protein database found in A *Trichonephila inaurata*, B *Phidippus regius*, C *Nuctenea umbratica*, or D *Avicularia* species respectively. Note that some of these hits do not represent full but only partial protein sequences. Moreover, since Starrett *et al.* have demonstrated that the known spidroin 2 sequence of *Avicularia juruensis* may potentially be an experimental artifact, we have excluded this spidroin from our analysis. [1] The provided data for *Avicularia avicularia* are not theoretical values, they were determined using the automated online derivatization method analogous to the values presented in the main text. The content of glutamic acid and glutamine, as well as aspartic acid and asparagine, were each combined into a single value.

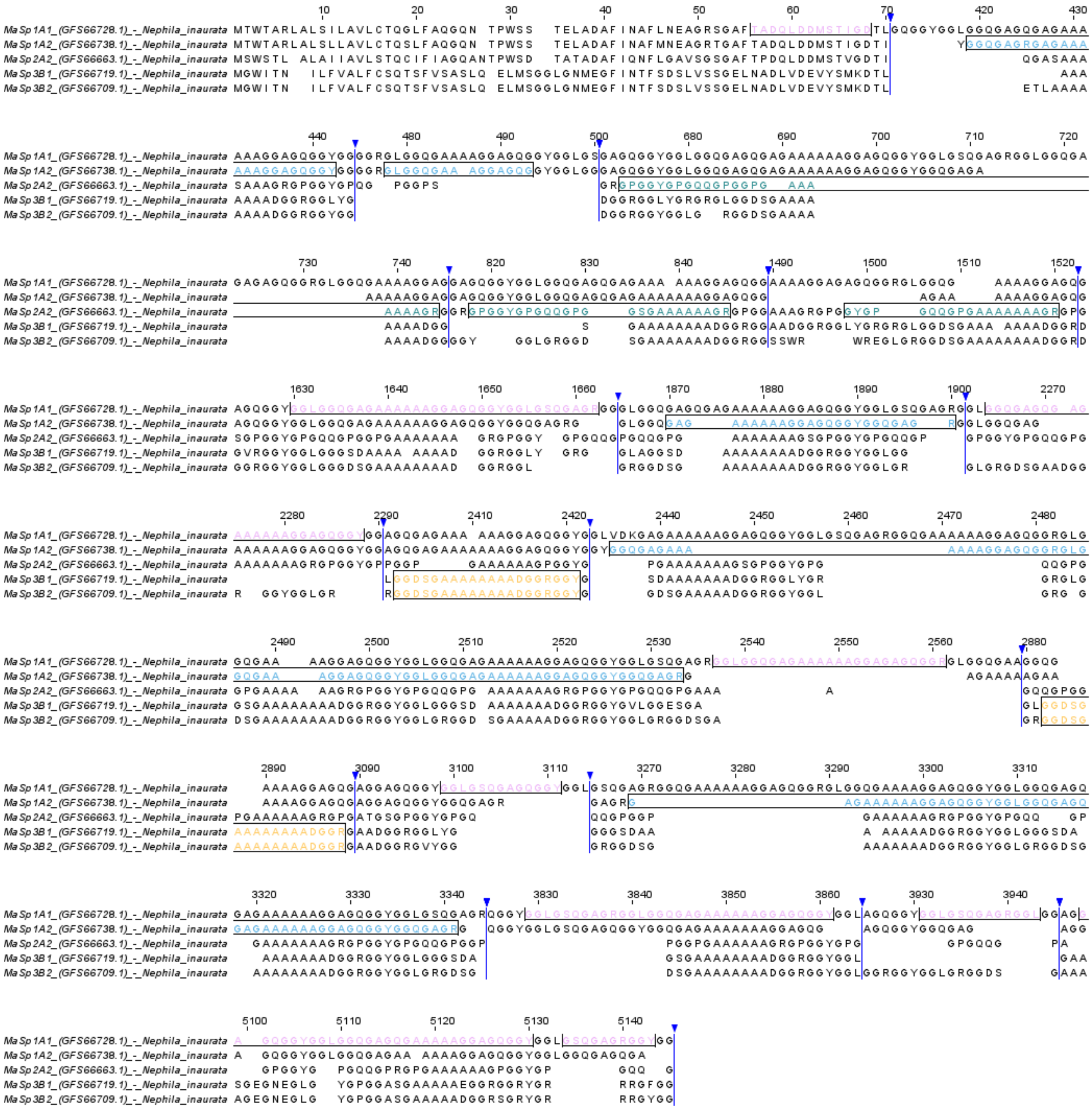

Figure S5: **Aligned amino acid sequences of spidroins identified in *Trichonephila inaurata* dragline silk by a search against the NCBI protein database, or de novo sequencing**. Regions of identity are marked with a black rectangle and a spidroin-specific font color. Vertical blue lines represent regions where parts of the amino acid sequence were removed as no alignment with detected peptides was achieved.

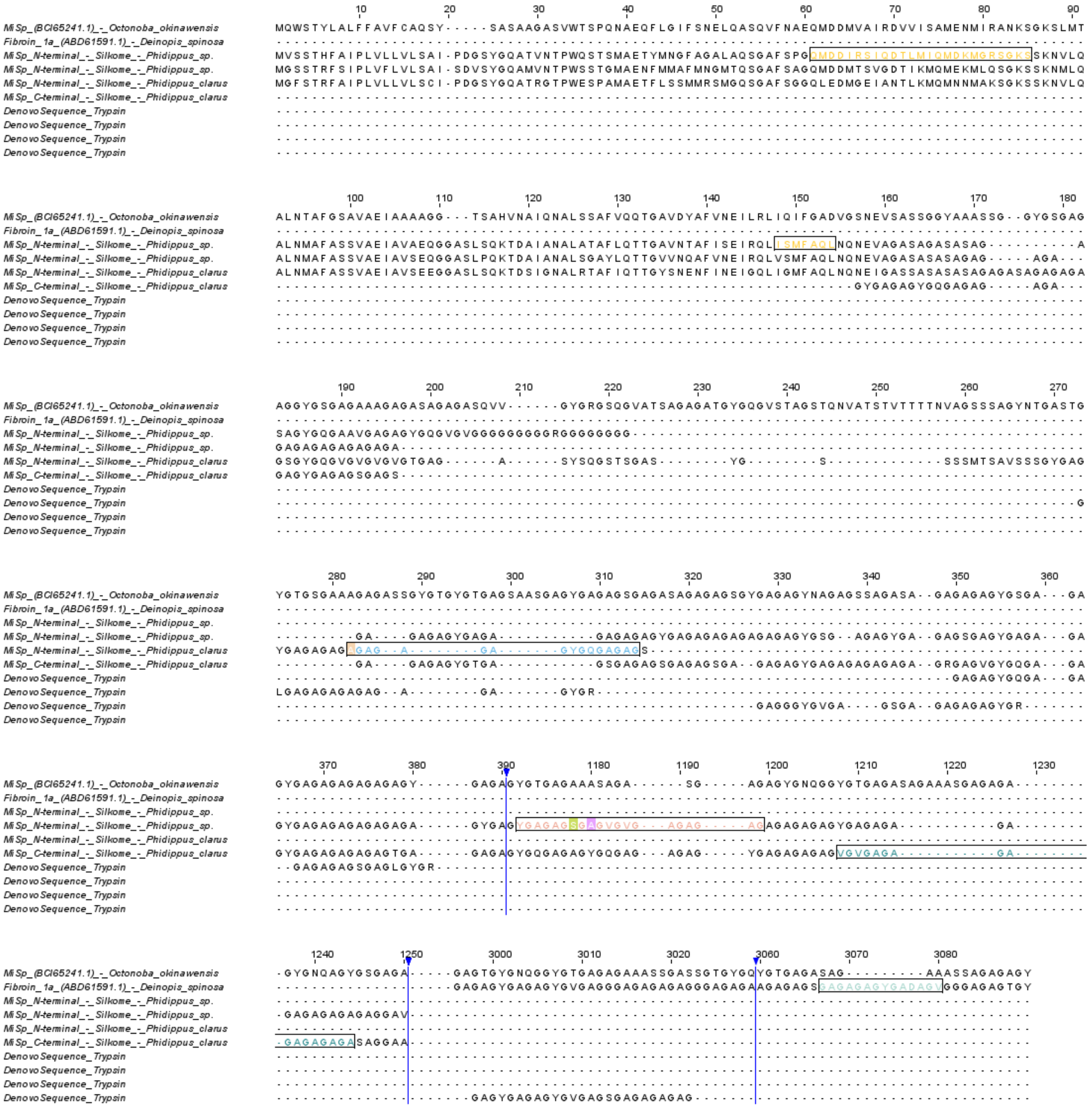

Figure S6: **Aligned amino acid sequences of spidroins identified in *Phidippus regius* silk by a search against the silkome and NCBI protein database, or de novo sequencing**. Regions of identity are marked with a black rectangle and a spidroin-specific font color. Text highlight color indicates the presence of a deviating amino acid of the identified peptide compared to the database (orange = Alanine (A) in the database, Valine (V) in sequence; green = Serine (S) in the database, Alanine (A) in sequence; pink = Alanine (A) in the database, Serine (S) in sequence). Vertical blue lines represent regions where parts of the amino acid sequence were removed as no alignment with detected peptides was achieved.

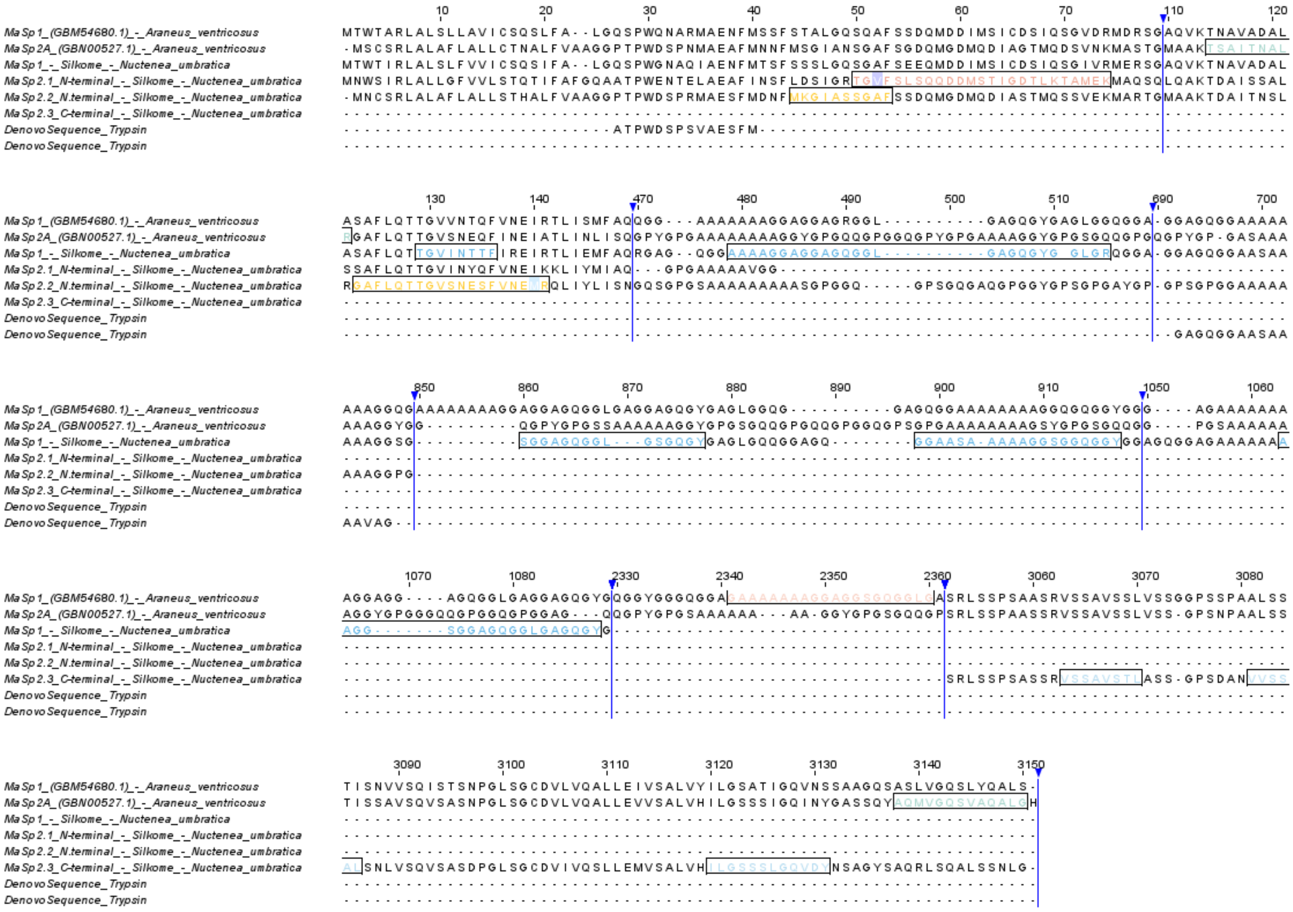

Figure S7: **Aligned amino acid sequences of spidroins identified in *Nuctenea umbratica* dragline silk by a search against the silkome and NCBI protein database, or de novo sequencing**. Regions of identity are marked with a black rectangle and a spidroin-specific font color. Text highlight color indicates the presence of a deviating amino acid of the identified peptide compared to the database (purple = Valine (V) in the database, Alanine (A) in sequence; light blue = Methionine (M) in the database, Isoleucine (I) in sequence). Vertical blue lines represent regions where parts of the amino acid sequence were removed as no alignment with detected peptides was achieved.

Table S1: Summary of results in the form of means and standard deviations.

Schwann Cell Characteristics			
Live Cell Imaging			
Group	Accumulated Velocity *[µm/min]*	Euclidean Velocity *[µm/min]*	Directness
Trichonephila inaurata	0.99 ± 0.09	0.19 ± 0.10	0.20 ± 0.09
Phidippus regius	1.21 ± 0.10	0.29 ± 0.10	0.24 ± 0.05
Nuctenea umbratica	0.87 ± 0.17	0.13 ± 0.07	0.15 ± 0.04
PLL-laminin Control	0.82 ± 0.15	0.12 ± 0.02	0.15 ± 0.03
Immunofluorescence			
Group	% of Proliferating Cells	% of Culture Purity	Nuclear Roundness *(1 = perfectly round)*
Trichonephila inaurata	19.21 ± 7.24	99.37 ± 0.67	0.74 ± 0.13
Phidippus regius	9.69± 2.55	99.50 ± 0.58	0.75 ± 0.11
Nuctenea umbratica	14.30± 5.27	99.71 ± 0.34	0.77 ± 0.12
PLL-laminin Control	63.10 ± 7.21	99.56 ± 0.34	0.60 ± 0.12

Material Properties		
Nanoindentation		
Group	Hardness [GPa]	Reduced Elastic Modulus [GPa]
Trichonephila inaurata	0.40 ± 0.05	6.82 ± 1.30
Phidippus regius	0.52 ± 0.07	9.73 ± 1.70
Nuctenea umbratica	0.41 ± 0.06	8.31 ± 0.94
Scanning Electron Microscopy		
Group	Diameter [µm]	
Trichonephila inaurata	6.05 ± 0.81	
Phidippus regius	2.19 ± 0.35	
Nuctenea umbratica	1.64 ± 0.03	

Table S2: **Identity of spidroins found in silk of *Phidippus regius* and spidroins that occur in *Trichonephila inaurata*, or *Nuctenea umbratica* silk.** Different spidroins identified in the three silks via mass spectrometry, and additional silks available in the NCBI or silkome protein database were selected for the alignment analysis via the constraint-based multiple alignment tool and the subsequent calculation of identity between the sequences.

	Sequence Identity [%]			
**Sequence**	MiSp N-terminal - Silkome [*Phidippus sp.*]	MiSp N-terminal - Silkome [*Phidippus sp.*]	MiSp N-terminal - Silkome [*Phidippus clarus*]	MiSp C-terminal - Silkome [*Phidippus clarus*]
MaSp1A1 (GFS66728.1) [*Trichonephila inaurata*]	49.5	43.6	40.2	49.4
MaSp1A2 (GFS66738.1) [*Trichonephila inaurata*]	47.6	44.7	41.8	50.0
MaSp2A2 (GFS66663.1) [*Trichonephila inaurata*]	46.2	37.8	39.5	39.8
MaSp3B1 (GFS66719.1) [*Trichonephila inaurata*]	27.9	31.3	26.9	34.8
MaSp3B1 (GFS66719.1) [*Trichonephila inaurata*]	27.9	32.5	28.3	36.6
MiSp1A1 (GFS66629.1) [*Trichonephila inaurata*]	40.7	45.6	43.4	51.5
MiSp1A2 (GFS66645.1) [*Trichonephila inaurata*]	49.6	49.8	46.6	53.9
MiSp1B1 (GFS66622.1) [*Trichonephila inaurata*]	46.9	46.7	42.2	44.7
MaSp1 (GBM54680.1) [*Araneus ventricosus*]	51.4	38.3	39.2	45.1
MaSp2A (GBN00527.1) [*Araneus ventricosus*]	45.9	35.5	41.6	36.6
MaSp1 N-terminal - Silkome [*Nuctenea umbratica*]	48.4	37.0	41.0	41.0
MaSp2.1 N-terminal - Silkome [*Nuctenea umbratica*]	47.6	39.7	39.6	26.2
MaSp2.2 N-terminal - Silkome [*Nuctenea umbratica*]	43.5	35.9	44.3	35.2
MaSp2.3 C-terminal - Silkome [*Nuctenea umbratica*]	27.4	42.6	22.1	36.7
MiSpA (GBM96470.1) [*Araneus ventricosus*]	34.4	46.4	36.5	56.1
MiSpB (GBM96188.1) [*Araneus ventricosus*]	33.3	45.4	37.5	56.7
MiSp2.3 N-terminal - Silkome [*Nuctenea umbratica*]	34.3	48.2	36.2	52.6

Table S3: List of primary and secondary antibodies

Primary Antibodies			
**Antigen**	**Species**	**Dilution**	**Company**
S100	rabbit	1:200	DAKO
SOX10	mouse	1:50	Santa Cruz
**Secondary Antibodies**			
**Fluorophore**	**Target Species**	**Dilution**	**Company**
AF488P	rabbit	1:600	Invitrogen
AF647	mouse	1:300	ThermoFisher

References

[1] J. Starrett, JE. Garb, A. Kuelbs, UO. Azubuike, CY. Hayashi, *PLoS One* **2012**, 7, 6.
